# Nitrogen cycling microbiomes are structured by plant mycorrhizal associations with consequences for nitrogen oxide fluxes in forests

**DOI:** 10.1111/gcb.15439

**Published:** 2020-12-15

**Authors:** Ryan M. Mushinski, Zachary C. Payne, Jonathan D. Raff, Matthew E. Craig, Sally E. Pusede, Douglas B. Rusch, Jeffrey R. White, Richard P. Phillips

**Affiliations:** ^1^ School of Life Sciences University of Warwick Coventry UK; ^2^ O'Neill School of Public and Environmental Affairs Indiana University Bloomington IN USA; ^3^ Department of Chemistry Indiana University Bloomington IN USA; ^4^ Department of Biology Indiana University Bloomington IN USA; ^5^ Environmental Sciences Division and Climate Change Science Institute Oak Ridge National Laboratory Oak Ridge TN USA; ^6^ Department of Environmental Sciences University of Virginia Charlottesville VA USA; ^7^ Center for Genomics and Bioinformatics Indiana University Bloomington IN USA; ^8^ Department of Earth and Atmospheric Sciences Indiana University Bloomington IN USA

**Keywords:** forest soils, metagenomes, mycorrhizae, nitrogen cycle, nitrous oxide, reactive nitrogen oxides

## Abstract

Volatile nitrogen oxides (N_2_O, NO, NO_2_, HONO, …) can negatively impact climate, air quality, and human health. Using soils collected from temperate forests across the eastern United States, we show microbial communities involved in nitrogen (N) cycling are structured, in large part, by the composition of overstory trees, leading to predictable N‐cycling syndromes, with consequences for emissions of volatile nitrogen oxides to air. Trees associating with arbuscular mycorrhizal (AM) fungi promote soil microbial communities with higher N‐cycle potential and activity, relative to microbial communities in soils dominated by trees associating with ectomycorrhizal (ECM) fungi. Metagenomic analysis and gene expression studies reveal a 5 and 3.5 times greater estimated N‐cycle gene and transcript copy numbers, respectively, in AM relative to ECM soil. Furthermore, we observe a 60% linear decrease in volatile reactive nitrogen gas flux (NO*_y_* ≡ NO, NO_2_, HONO) as ECM tree abundance increases. Compared to oxic conditions, gas flux potential of N_2_O and NO increase significantly under anoxic conditions for AM soil (30‐ and 120‐fold increase), but not ECM soil—likely owing to small concentrations of available substrate (${\text{NO}}_{3}^{‐}$) in ECM soil. Linear mixed effects modeling shows that ECM tree abundance, microbial process rates, and geographic location are primarily responsible for variation in peak potential NO_y_ flux. Given that nearly all tree species associate with either AM or ECM fungi, our results indicate that the consequences of tree species shifts associated with global change may have predictable consequences for soil N cycling.

## Graphical abstract

Volatile nitrogen oxides can negatively impact climate, air quality, and human health. Using soils collected from temperate forests across the eastern United States, we show microbial communities involved in nitrogen (N) cycling are structured, in large part, by the composition of overstory trees, leading to predictable N cycling syndromes, with consequences for emissions of volatile nitrogen oxides to air. Trees associating with arbuscular mycorrhizal (AM) fungi promote soil microbial communities with higher N‐cycle potential and activity, relative to microbial communities in soils dominated by trees associating with ectomycorrhizal (ECM) fungi. Given that nearly all tree species associate with either AM or ECM fungi, our results indicate that the consequences of tree species shifts associated with global change may have predictable consequences for soil N cycling.

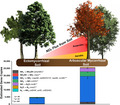

## 1 INTRODUCTION

One of the major pathways for nitrogen (N) loss in forest soil is through volatilization of reduced N (e.g., nitrogen oxides). Studies in North America have illustrated that anthropogenic sources of nitrogen oxides to air have declined significantly over the past two decades, including an estimated 4% decrease since 2005 (Jiang et al., 2018; Romer et al., 2018; Simon et al., 2015). However, increases in the use of N‐based fertilizers (Bouwman et al., 2002; Lu & Tian, 2017), warmer global temperatures (Hansen et al., 2010), and land‐use change (Jain et al., 2013; Wang et al., 2017) have increased the importance of non‐anthropogenic sources, such as soil, to the global N budget. Nitrous oxide (N_2_O) and the suite of volatile reactive nitrogen oxide compounds (NO*_y_* ≡ NO, NO_2_, HONO, etc.) are major products that have profound effects on climate and atmospheric chemistry. N_2_O is one of the most potent greenhouse gases with roughly 300 times the global warming potential of carbon dioxide (CO_2_), and has been shown to deplete stratospheric ozone (O_3_) (Finlayson‐Pitts & Pitts Jr., 2000). Additionally, NO*_y_* species drive photochemical ozone production, are precursors to acid rain (via HNO_3_), and influence the oxidizing capacity of the atmosphere by sustaining photocatalytic cycles of HO*_x_* (Bouwman et al., 2002; Steinkamp et al., 2009). While N emissions from agricultural systems are well characterized, significantly less is known about the sources and sinks of nitrogen oxides in natural ecosystems, such as upland forest soils, as well as the environmental properties and conditions that influence N_2_O and NO*_y_* flux.

**Figure 1:**
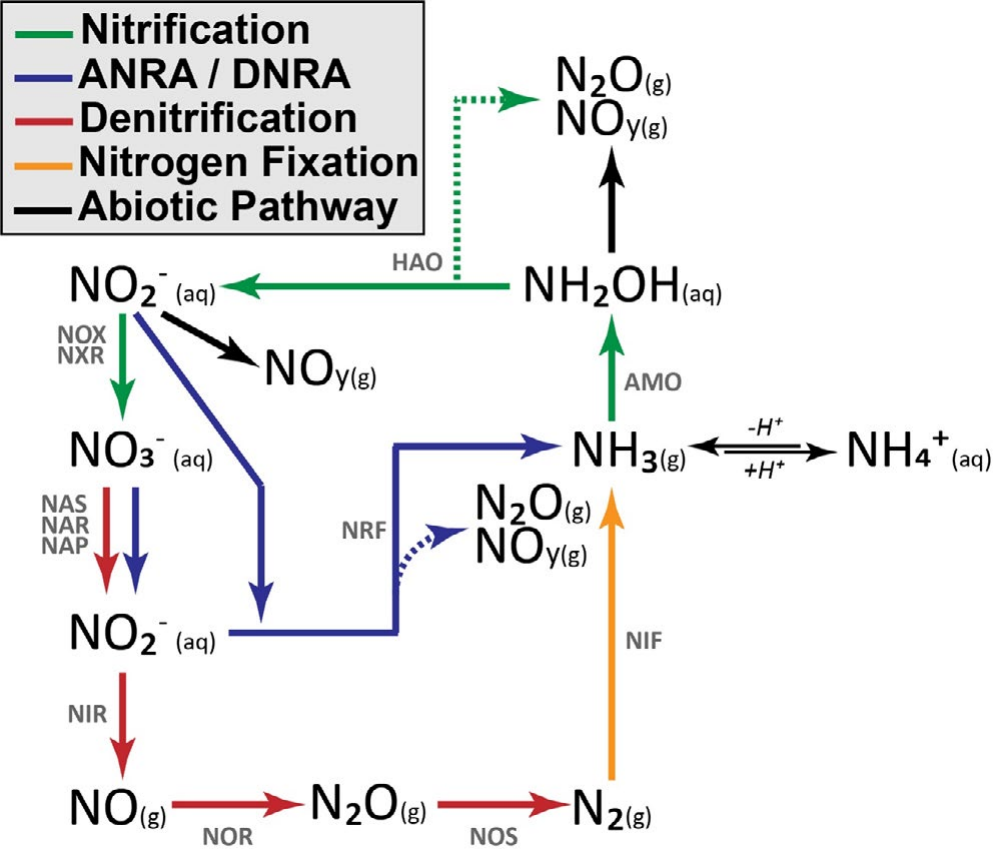
Simplified diagram showing the enzymatic steps for nitrification, assimilatory and dissimilatory nitrate reduction to ammonia (ANRA/DNRA), denitrification, and nitrogen fixation processes. Dashed lines indicate production of gaseous byproducts of enzyme activity. Enzymes are noted above pathways. AMO: ammonia monooxygenase; HAO: hydroxylamine oxidoreductase; NOX/NXR: nitrate oxidoreductase; NAS/NAR/NAP: nitrate reductase; NRF/NIR: nitrite reductase; NOR: nitric oxide reductase; NOS: nitrous oxide reductase; NIF: nitrogenase reductase

Microbial oxidation and reduction drive the vast majority of soil nitrogen oxide fluxes. The biological cycling of N_2_O and NO*_y_* can be directly linked to various microbial processes, primarily nitrification and denitrification but also assimilatory and dissimilatory nitrate reduction (ANRA/DNRA; Figure 1). The rate‐limiting step for nitrogen oxide production is generally considered to be associated with nitrification, which is defined as a cascade of oxidative metabolism from ammonia to nitrate. This process can be identified by the activities of ammonia‐oxidizing archaea (AOA) and bacteria (AOB) as well as nitrite‐oxidizing bacteria (NOB), which have all been linked to N_2_O and NO*_y_* production under oxic conditions (Mushinski et al., 2019; Robertson & Tiedje, 1987; Scharko et al., 2015). Specifically, nitrification‐derived NO*_y_* and N_2_O have been correlated to the abundance of nitrification genes and transcripts (Soares et al., 2016). The most common targets include genes associated with ammonia monoxygenase (AMO), hydroxylamine oxidoreductase (HAO), and nitrite oxidoreductase (NXR). Although aerobic nitrifiers are considered rate limiting and can contribute to fluxes of N_2_O and NO*_y_*, most emissions are thought to be a function of denitrifying heterotrophic microbes, albeit through processes associated with reducing conditions such as assimilatory and dissimilatory nitrate reduction, as well as the multi‐step reduction of nitrite to N_2_ (Ambus & Zechmeister‐Boltenstern, 2007). Microbial groups associated with denitrification are highly diverse, but their relative abundance and activity can be determined by quantifying the abundance of gene and transcript copies associated with reduction of nitrate (NAR/NAP), nitrite (NIR), nitric oxide (NOR), and nitrous oxide (NOS) (Levy‐Booth et al., 2014). Reducing conditions lead to upregulation of these denitrification genes, a situation commonly found in anoxic soil microsites, resulting in hotspots of N_2_O and NO*_y_* flux (Kuzyakov & Blagodatskaya, 2015). Therefore, there is a need to quantify nitrogen oxide fluxes and the associated microbial responses to varying oxygen conditions.

Forest soil microbial communities are strongly influenced by vegetation composition (Lladó et al., 2017). Considering that forests are currently experiencing large‐scale shifts in tree species composition (Jo et al., 2019), due to natural and anthropogenic effects such as climate change, atmospheric deposition, alterations in disturbance, habitat fragmentation, and exotic species invasion, there is a need to develop robust frameworks for investigating the microbial and biogeochemical consequences of such changes. Forests within the United States are comprised of a mixture of tree species that associate primarily with either arbuscular mycorrhizal fungi (AM) or ectomycorrhizal fungi (ECM). Examples of AM tree genera include maple (*Acer*), tulip (*Liriodendron*), cherry (*Prunus*), and ash (*Fraxinus*) while ECM tree genera include oak (*Quercus*), hickory (*Carya*), and beech (*Fagus*). Given that AM and ECM trees possess different nutrient use traits (Beidler et al., 2020; Cheeke et al., In press; Keller & Phillips, 2019; Lin et al., 2017) and promote unique soil microbial assemblages (Cheeke et al., 2017), the relative abundance of AM or ECM trees in a stand may be an effective integrator of various ecosystem processes (Phillips et al., 2013). Researchers have used the AM and ECM categorization to reflect distinct biogeochemical syndromes (e.g., AM: inorganic nutrient economy, fast N cycling; ECM: organic nutrient economy, slow N cycling). Consequently, “mycorrhizal gradients” (defined as plots varying in their relative abundance of AM vs. ECM trees within an ecosystem) have been proposed as an appropriate experimental “proving ground” for exploring the effect of species shifts on biogeochemical processes in these forest ecosystems (Jo et al., 2019).

Numerous studies have observed that AM and ECM forests differ with respect to N cycling (Craig et al., 2018; Lin et al., 2017; Midgley & Phillips, 2014; Zhu et al., 2018). AM soils tend to possess low C:N ratios, large pools of inorganic N, and high rates of nitrification and are generally referred to as having “open” N cycles (e.g., high N loss relative to N recycled); conversely, ECM soils possess wide C:N ratios, large pools of organic N, and low rates of net nitrification and are referred to as having “closed” N cycles. Read (1991) first illustrated differences between AM and ECM soil N cycling, hypothesizing that ECM soils have lower nitrogen mineralization rates (relative to AM soils) owing to the large amounts of N bound to soil organic matter. More recently, Phillips et al. (2013) hypothesized that the abundance of AM and ECM trees in a plot, stand, or region may provide an integrated index of N cycling. The framework proposed by Phillips is supported by a recent meta‐analysis (Lin et al., 2017), which used a global dataset to illustrate that inorganic N concentrations, net N mineralization, and nitrification rates are all higher in AM relative to ECM forests; however, we have a limited understanding of the underlying microbial contributions to the patterns, and the consequences for N retention and loss. Furthermore, the high rates of N‐cycle activity in AM soil, under oxic conditions, may be exacerbated as soil becomes more anoxic. Thus, comparing N‐gas fluxes and microbial activity under a factorial combination of oxic and anoxic conditions in AM and ECM soil will help better define these hotspots and hot moments in forest soils.

Recently, Mushinski et al. (2019) reported that AM soils produce significantly higher fluxes of NO*_y_* under oxic conditions, relative to ECM soils at a single site. However, the following questions remain: To what extent do microbial communities involved in N cycling differ across mycorrhizal gradients within temperate forests, what are the influences of oxic and anoxic conditions on N‐cycle processes in AM and ECM soils, and what environmental variables best explain N fluxes in AM and ECM forests? In this study, we used metagenomic analyses and microcosm experiments to investigate the potential for gaseous emissions of nitrogen oxides in AM‐ and ECM‐dominated soils as well as along a gradient of AM to ECM soils throughout the eastern United States. Additionally, the response of key microbial transcripts coding for enzymes AMO, NIR, and NOS in AM‐ and ECM‐dominated plots were analyzed by reverse transcription quantitative PCR (RT‐qPCR). We hypothesized that (a) forests with more “open” N cycles (e.g., AM‐dominated stands) contain microbial communities with greater numbers of N‐cycling taxa and genes that are specifically related to the production of volatile nitrogen oxides, (b) anoxic soil conditions lead to significant increases in nitrogen oxide production and transcript copy numbers of denitrification genes in both AM and ECM soil; however, fluxes will be higher in AM soil relative to ECM soil, and (c) the combined effects of N‐cycle process rates and the relative abundance of N‐cycle taxa will explain a significant amount of variation in the peak flux of soil NO_y_ and N_2_O.

## 2 MATERIALS AND METHODS

### 2.1 Site description

**Figure 2:**
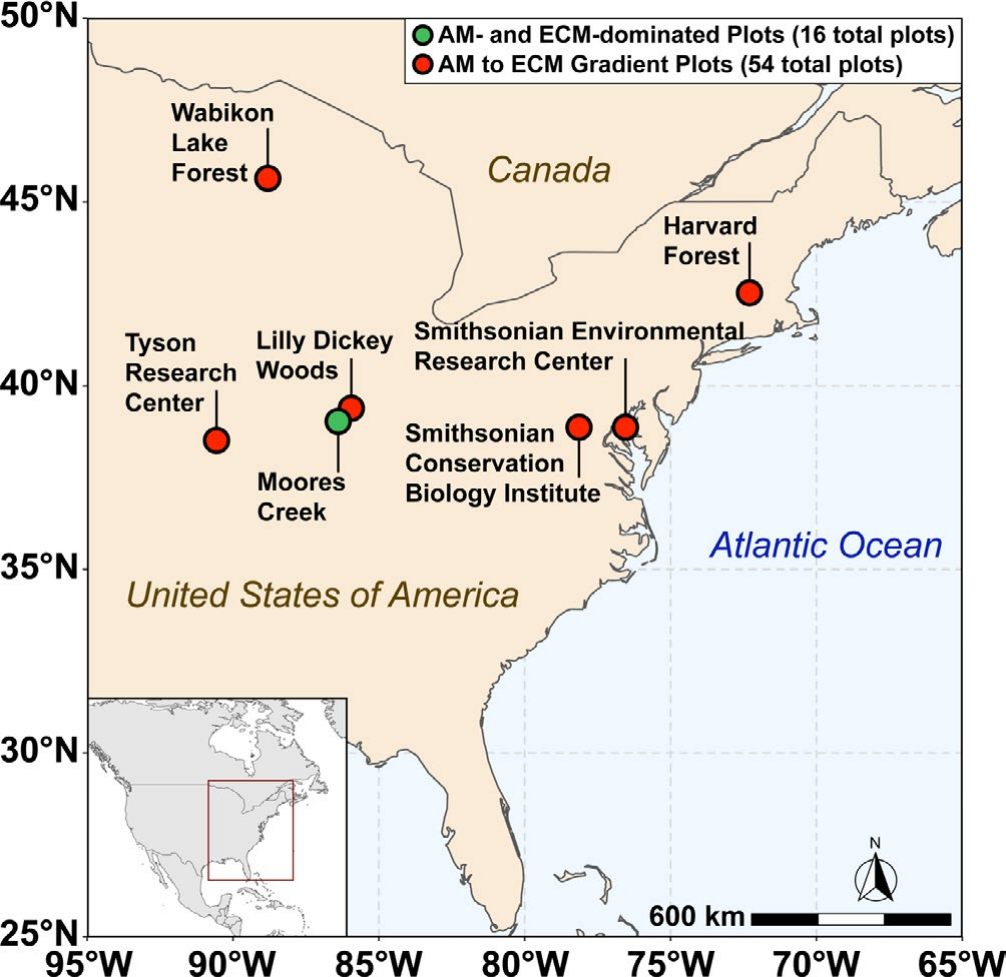
Map of sampling locations across the eastern United States used in this study. The green dot indicates Moores Creek where AM and ECM dominated plots are located while the red dots indicate sites associated with the 54‐plot gradient. The Moores Creek site contains 16 total plots (8‐AM and 8‐ECM dominated) while each site in the 54‐plot gradient contains nine plots of varying AM/ECM composition

Soils were collected in August 2017 from eight AM‐ and eight ECM‐dominated plots (dominance implies >85% of the basal area of the plot) at the Indiana University Research and Teaching Preserve. This site will herein be referred to as “Moores Creek.” Each plot at Moores Creek was 20 m × 20 m. Additionally, soil was sampled in the summer of 2019 from a 54‐plot AM to ECM gradient throughout the eastern United States—herein referred to as “gradient plots.” The gradient plots of six sites, each containing nine 20 m × 20 m plots that varied the relative proportion of AM and ECM trees (e.g., from 0% to 100% AM/ECM tree abundance at each site). Gradient plots were located within research forests of the Smithsonian ForestGEO network (https://forestgeo.si.edu/sites‐all). For both Moores Creek and the gradient plots, trees were categorized by mycorrhizal association, and the percent of ECM trees (based on basal area) was determined according to Phillips et al. (2013). Site locations for plots used in this study can be found in Figure 2. Dominant soil orders in this study are inceptisols [Moores Creek (MC) and Lilly Dickey Woods (LDW)], alfisols [Smithsonian Conservation Biology Institute (SCBI)], ultisols [Smithsonian Environmental Research Center (SERC)], spodosols [Harvard Forest (HF) and Wabikon Lake Forest (WLF)].

### 2.2 Soil sampling

Within each plot (both Moores Creek and gradient plots), five soil cores (5.08 cm diameter) were collected at 0–5 cm. These individual soil cores were pooled by plot to increases mass and reduce environmental heterogeneity, resulting in 16 composited samples for Moores Creek and 54 composited samples for the gradient plots. From each composited sample, approximately 5 g of soil was immediately subsampled for molecular analysis and suspended in a preservation buffer (DNA/RNA Shield, Zymo Research), then shipped back to Indiana University whereupon the preservation buffer was removed, and soil was stored at −80°C. The remaining composited sample was transported on ice packs to Indiana University where a 40 g aliquot of soil was dried at 105°C for gravimetric moisture and a 25 g aliquot was air‐dried for soil pH analysis. The remaining field‐moist soil was passed through a 2 mm sieve to further homogenize the soil, and to remove large organic matter fragments and rocks. Sieved soil was then stored at 4°C until further analysis.

### 2.3 Overview of laboratory analyses

Soils from both Moores Creek and the gradient plots were analyzed for a common suite of physicochemical properties, including soil carbon and nitrogen content, soil pH, net nitrogen mineralization rate, and net nitrification rate. Gas fluxes (CO_2_, NO*_y_* and N_2_O) were also analyzed for both sets of soils using microcosm incubations. Additionally, concentrations of oxalate‐extractable iron and particle size analysis were analyzed for gradient plot soil. These physicochemical properties and gas flux measurements were used primarily to explore *hypothesis 1* and *3*, where we define gas fluxes in relation to microbial community composition and which soil factors explain the majority of variation in NO*_y_* and N_2_O flux. DNA extracted from both Moores Creek and gradient plot soil was subjected to shotgun metagenomic sequencing; however, sequencing depth differed between the two groupings. Moores Creek soil DNA was used for deep shotgun sequencing (200 million reads per sample) whereas gradient plot soil DNA was used for a shallower analysis (12 million reads per sample). Both of these datasets were used to address *hypothesis 1* and *3*. Moores Creek sieved soil was used to determine the relative influence of oxic and anoxic conditions on AM and ECM soil; specifically, the N‐cycle microbial response, N‐cycle rates, and fluxes of N gases. This set of experiments addresses *hypothesis 2* where we predict that anoxic conditions will stimulate N‐cycle processes in AM soil more so than ECM soil. Detailed procedures are noted in the subsequent paragraphs.

### 2.4 Soil physicochemical analysis

Soil pH was measured using an Orion pH meter (ThermoFisher Scientific, Waltham, MA, USA) on a 1:2 solution of air‐dried soil in a 0.01 M CaCl_2_ solution. A 40 g sample of soil was dried at 105°C for 48 hr to calculate bulk density and gravimetric water content (GWC) as described in Mushinski et al. (2019). Ten grams of sieved soil was dried at 60°C for 48 hr, ground to a powder, and analyzed for soil total carbon (TC) and nitrogen (TN) using a Costech ECS 4010 elemental analyzer (Costech Analytical Technologies Inc.). Nitrate (${\text{NO}}_{3}^{‐}$) pools were quantified from 4 g of sieved, field‐moist soil with 15 ml of 2 M KCl within 36 hr of soil being taken from the ground and analyzed using a Lachat QuikChem 8000 Flow Injection Analyzer (Lachat Instruments). Total net nitrification and mineralization rates were calculated as the accumulation or depletion of inorganic N (mineralization: ${\text{NH}}_{4}^{+}+{\text{NO}}_{3}^{‐}$; nitrification: ${\text{NO}}_{3}^{‐}$) over the course of a 14 day incubation. Soil texture was determined using a standard hydrometer procedure (Ulmer et al., 1994). Some plots had extremely high organic matter content in 0–5 cm depth increments, so plot level soil texture was derived from 5 to 15 cm increments. We quantified oxalate‐extractable Al and Fe pools in all soil samples as an index of poorly crystalline Al‐ and Fe‐oxides (Schwertmann, 1973); specifically, 0.40 g air dried pulverized soil was suspended in 40 ml 0.2 M ammonium‐oxalate at pH 3.0 in the dark for 4 hr, gravity filtered, and analyzed with an atomic‐absorption spectrometer (AAnalyst 800, Perkin Elmer), using an acetylene flame and a graphite furnace for the atomization of Fe and Al, respectively.

### 2.5 Measurement of soil gas fluxes under oxic conditions

All soils (normalized to 40% gravimetric water content) were preincubated in 100 mm diameter polystyrene petri dishes, in the dark for 24 hr at 20°C. Following soil preincubation, the petri dishes were loaded into 2 L glass jars, herein referred to as *chambers*, which were capped with polytetrafluoroethylene (PTFE) lids containing PTFE inlet and outlet ports. All glass and polystyrene surfaces were coated with an inert perfluorinated polymer film (Fluoropel PFC 801A, Cytonix Corp.) and Teflon tubing and PTFE fittings were used to reduce adsorption of reactive nitrogen oxides that could interfere with measurements. During incubation, the flux chambers were covered in black cloth to prevent photochemical conversions of reactive nitrogen oxides on soil surfaces.

Each experiment utilized five flux chambers; one contained an empty petri dish to act as the blank and four contained a petri dish with soil. During the course of the incubation, ultrapure zero‐air was continuously flowed through a 5 Å molecular sieve and into a six‐way PTFE manifold, delivering a continuous flow through each chamber [2 L/min, 1 atm, 0% relative humidity (RH)]. A solenoid array controlled by a microprocessor (R2 Series, OPTO 22) selectively sampled the outflow of gas from each of the chambers during their measurement period over the course of the 48 hr experiment cycle. Gas flow from each soil‐containing chamber was selectively sampled continuously for 10 min each hour, whereupon concentrations of CO_2_, N_2_O, NO, NO_2_, and HONO were analyzed. Measurements of the blank chamber flanked each soil measurement time period and were averaged over the course of each hour to determine background trace gas concentrations used in flux calculations.

Nitric oxide fluxes were measured using a modified chemiluminescence NO_x_ analyzer (Air Quality Design, Inc.). The chemiluminescence instrument was equipped with a photolytic cell containing two LEDs with peak wavelengths at 385 and 395 nm. This allowed for the measurement of nitrogen dioxide (NO_2_) and nitrous acid (HONO) fluxes through differential photolysis (Reed et al., 2016). N_2_O fluxes were measured using an LGR ICOS (off‐axis integrated cavity output spectroscopy) N_2_O/CO Analyzer (Los Gatos Research Inc.). Soil water content was calculated from air moisture concentration using linear regression of known gravimetric water content (GWC) at time 0 and 48 hr. Specifically, water vapor concentration of each chamber was regressed against actual GWC at time 0 hr (40% GWC) and 48 hr (0% GWC) to define an equation for GWC estimation based on water vapor. The equation was verified by measuring GWC at various time points between 0 and 48 hr. Carbon dioxide fluxes were measured using a LI‐COR CO_2_/H_2_O analyzer (LI‐840 A, LI‐COR Inc.) and were used as a proxy for microbial respiration.

To calculate flux, concentrations of CO_2_, N_2_O, NO, NO_2_, and HONO were compared to a blank chamber using Equation (1).

(1) $$\text{Flux}=\frac{1}{\tau }\times \frac{F_{\text{tot}}\left(C_{\text{soil}}‐C_{\text{blank}}\right)}{m_{\text{soil}}}$$

In Equation (1), τ is the residence time of gas in the chamber, *F_tot_* is the flow of the carrier gas, *m_soil_* is the mass of soil, and *C_soil_* and *C_blank_* are the concentrations of analyte gas measured in the soil‐containing and blank chambers, respectively. Positive fluxes describe net transfer of gases from soil to air (outgassing), while negative fluxes represent net transfer from air to soil (i.e., deposition or consumption). Flux data used for regression analysis were the peak flux value from each 48 hr experiment. NO*_y_* fluxes are defined as the combined fluxes of NO, NO_2_, and HONO.

### 2.6 DNA extraction, shotgun sequencing, and metagenomic analysis

Moores Creek soil was used for deep shotgun sequencing (200 million reads per sample) and subsequent metagenomic analysis. This depth of sequencing has been shown to provide accurate representation of the relative counts of genes for a given sample (Gweon et al., 2019). For all 16 samples, soil DNA was extracted from 0.3 to 0.4 g field‐moist soil using a DNeasy PowerSoil Kit (Qiagen) and then six of the extracts were selected for downstream analysis using a random number generator. The purity of subsampled DNA extracts was measured using an Epoch microplate spectrophotometer (BioTek) and monitoring the absorbance (*A*_λ_) at *λ* = 260 and 280 nm. Mean *A*_260_/*A*_280_ ratios were 1.94 ± 0.04 (mean ± *SD*), indicating that protein and/or residual reagent contamination was minimal. Extracts were further verified by gel electrophoresis where no RNA bands were observed. Roughly 3 μg of extracted DNA per sample was then sent to the DOE Joint Genome Institute (https://jgi.doe.gov/) for library preparation and shotgun sequencing on an Illumina NovaSeq 6000. Resulting raw Illumina reads were trimmed, quality filtered, and corrected using BFC (version r181) with the following options: −1 ‐s 10g ‐k 21 ‐t 10 (Li, 2015). Reads were then assembled using SPAdes assembler 3.12.0 using the following options: ‐m 2000–only assembler –k 33,55,77,99,127–meta –t 32 (Nurk et al., 2017). The entire filtered read set was mapped to the final assembly and coverage information generated using bbmap (version 38.22) using default parameters except ambiguous = random (https://bbtools.jgi.doe.gov). The version of the processing pipeline was *jgi_meta_run.py* (version 2.0.1). The assembly pipeline resulted in 1.95 × 10^8^ aligned reads per sample with no significant difference between AM and ECM samples. Aligned reads were then subjected to the IMG/M pipeline where estimated gene copies associated with N‐cycle activity within the Kegg Pathway (KO) database (https://www.genome.jp/kegg/pathway.html) were quantified. Estimated gene copy numbers were calculated as the number of genes multiplied by the average coverage of the contigs, on which these genes were predicted (Huntemann et al., 2016). Table S1 defines the specific KO associated with this analysis. Statistical differences between the number of N‐cycle genes within AM and ECM metagenomes was assessed using Fisher's exact test.

N‐cycle taxa distribution and functional potential was analyzed using DNA from the gradient plots. This was done to determine if N‐cycle microbial trends seen in AM‐ and ECM‐dominated plots were consistent across a large spatial gradient. Soil DNA from 54 plots of varying AM and ECM aboveground composition was extracted and quality‐checked using the same methods mentioned above. Shotgun sequencing of extracted DNA took place at Indiana University's Center for Genomics and Bioinformatics (https://cgb.indiana.edu/) on an Illumina Hi‐Seq platform, resulting in 1.16 × 10^7^ reads per sample and a mean quality score (Q‐score) of 34.6 ± 0.1 (mean ± *SD*). Raw FASTQ files were analyzed using the MG‐RAST pipeline where they were initially dereplicated to remove sequence artifacts (Gomez‐Alvarez et al., 2009), screened to remove contaminant reads (Langmead et al., 2009), and trimmed to remove low‐quality sequences using DynamicTrim at a minimum phred score of 15 (Cox et al., 2010). Resulting reads were taxonomically and functionally annotated against NCBI’s RefSeq and KEGG’s KO database, respectively. Data were further screened to include only N‐cycle genes (KO) and associated taxa. N‐cycle reads were normalized as a relative abundance value per the total number of N‐cycle functional or taxonomic reads per sample.

### 2.7 AM and ECM soil response to anoxic conditions

Using Moores Creek soil, two analytical aliquots per sample (30 g) were placed in petri dishes and normalized to 40% GWC, which corresponded 68 ± 16% water filled pore space (WFPS). Samples were then pre‐incubated in the dark at 23°C for 24 hr. Following pre‐incubation, the first analytical replicate was placed into a sampling chamber, noted previously, whereupon ultrapure zero‐air was continuously flowed throughout the chamber (2 L/min, 1 atm, 0% relative humidity) for 24 hr and concentrations of CO_2_, N_2_O, and NO were continuously quantified from the outflow of gas. The second analytical replicate was placed in the same sampling chamber, but instead of air, ultrapure N_2_ (>99% N_2_) was flowed through the system for 24 hr to simulate anoxic conditions. Measurements of gases from the anoxic replicate were performed in an identical manner to the oxic replicate. By the end of the experiment, all soils had reached 22 ± 3% GWC. To calculate flux, concentrations of CO_2_, N_2_O, and NO for the oxic and anoxic samples were compared to a blank chamber using Equation (1).

Net nitrification was also assessed under oxic and anoxic conditions. Specifically, 10 g of sieved, field fresh Moores Creek soil was weighed out, in duplicate, into 125 ml Wheaton bottles and capped with airtight butyl stoppers. One replicate (oxic) was flushed with ultra‐pure air (20% O_2_, 80% N_2_) for ~ 1 min, while air from the other replicate was evacuated and replaced with helium (anoxic). Fresh air or helium was flushed through the incubations every 24 hr; field‐level soil moisture was sustained throughout the experiment. Samples were incubated in the dark for 14 days and then soil was extracted with 25 ml of 2 M KCl, whereupon ${\text{NO}}_{3}^{‐}$ was quantified as noted previously.

To determine microbial N‐cycle gene expression under different levels of oxygen availability, AM‐ and ECM‐dominated soils from Moores Creek were incubated under different headspace atmospheres [oxic: ultra‐pure air (20% O_2_, 80% N_2_) or anoxic: helium (100% He)] for 8 and 24 hr. These time points were selected because they corresponded to the observed peak flux (8 hr) and the end of incubation (24 hr) for the gas flux experiment. Specifically, four analytical replicates (10 g) of each soil were placed in 125 ml Wheaton bottles. Two of the replicates were flushed with ultra‐pure air, while the other two were flushed with helium. Soils were then incubated in the dark for the allotted time. At 8 hr after the initiation of the experiment, one oxic and one anoxic replicate were destructively sampled and ~0.5 g soil was used for RNA extraction. This was also done at 24 hr after the initiation of the incubation. Soil RNA was extracted using the RNeasy PowerSoil Total RNA Kit (Qiagen) and further purified with the RNase‐Free DNase Set (Qiagen). To quantify transcript abundance, cDNA was synthesized from the purified RNA with the QuantiTect Reverse Transcription Kit (Qiagen). Quantitative PCR (qPCR) of cDNA was performed using SsoAdvanced^TM^ Universal SYBR^®^ Green Supermix (Bio‐Rad) on a QuantStudio 7 Flex Real‐Time PCR System (ThermoFisher Scientific). Each plate included three analytical replicates per biological sample, synthetic oligionucleotide standards, and negative controls, also in triplicate. Copy numbers for each biological replicate were the average value of the three analytical replicates. Information on primers and thermocycling parameters is provided in Table S2.

### 2.8 Statistical analyses

Soil physicochemical and gas flux data were analyzed using a mixed effects model where ectomycorrhizal tree abundance was the fixed effect and geographic location was the random effect. Data for N‐cycle taxonomy from all soils were visualized using phylum percentage plots and differences in relative abundances were assessed using a two‐factor ANOVA where independent variables included plot location and mycorrhizal category (Table S3). The mycorrhizal category included three levels grouped into AM‐soil (plots ≥ 85% AM tree species), mixed‐soil (<85% AM and ECM tree species), and ECM‐soil (plots ≥ 85% ECM tree species). Functional distribution of N‐cycle genes was visualized using a non‐metric multidimensional scaling (NMDS) plot, based on normalized Bray‐Curtis distance matrices, where individual genes were grouped into process groupings. Significant physicochemical properties (*p* < .05) were included as vectors on the NMDS. All analyses were done in R, using the *anova_test* package for ANOVA and *vegan* for the NMDS. Significant differences among gas fluxes under oxic and anoxic conditions from Moores Creek soil were analyzed with a two‐way repeated measures ANOVA, where stand‐type (AM or ECM) and incubation headspace atmosphere [oxic: ultra‐pure air (20% O_2_, 80% N_2_) or anoxic: dinitrogen (>99% N_2_)] were fixed effects and incubation time (each hour for gas flux) was included as a repeated measure using the *anova_test* function in R and visualized using OriginPro 2018. Transcript copy number were analyzed with the same two‐way ANOVA procedure noted above. To assess the relative importance of vegetation composition, soil physicochemical properties, N‐cycle process rates, and N‐cycle microbes on peak flux values of N_2_O and NO_y_, we used a mixed effects model where the abundance of ECM trees, soil C:N, soil pH, percent clay, oxalate extractable iron, and aluminum, the relative abundance of N fixers, nitrifiers, and denitrifiers were fixed effects and geographic location (sampling sites) were random effects. This analysis was done with the *lme4* package and visualized with the *sjPlot* package, all in R.

## 3 RESULTS

### 3.1 Metagenomic analysis

**Figure 3:**
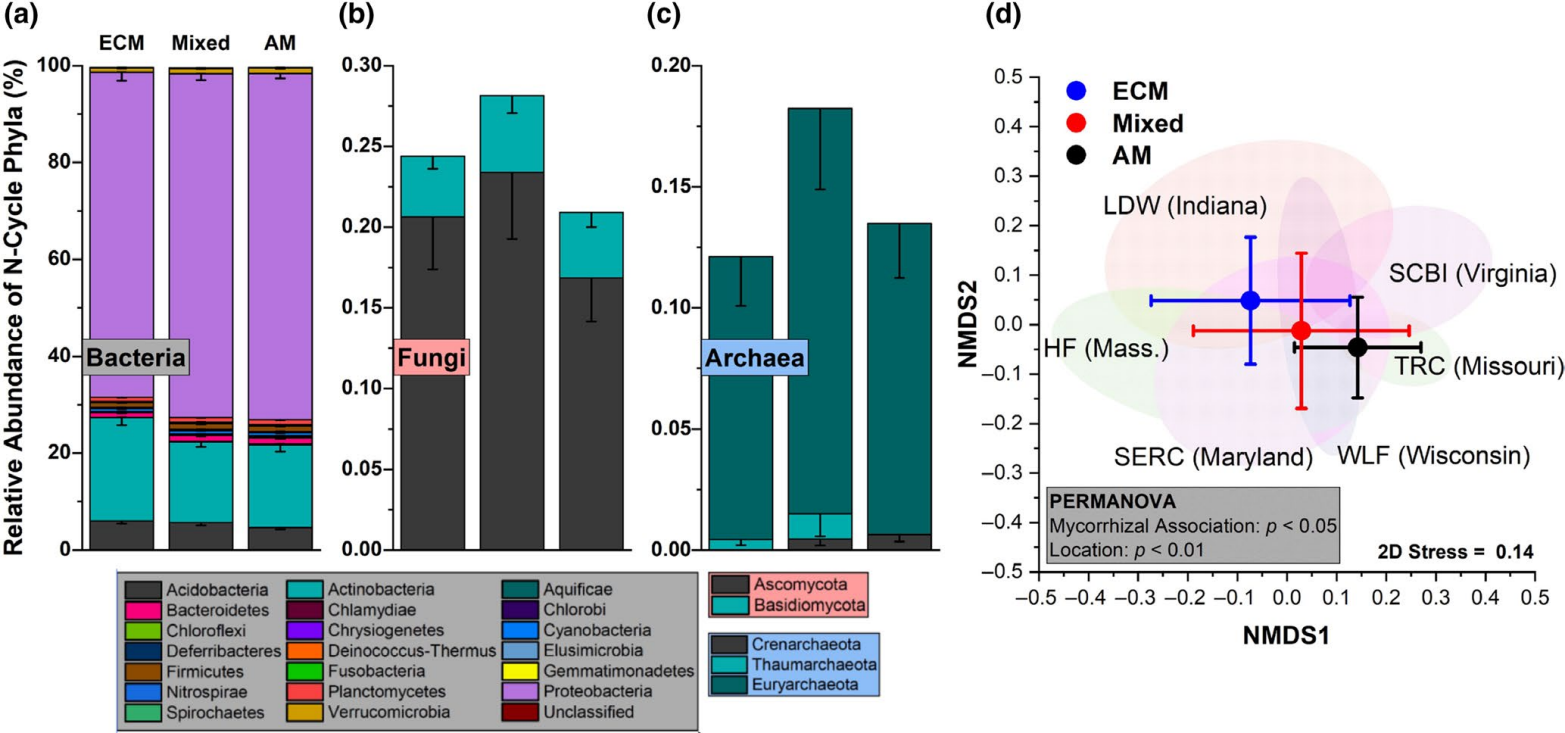
Relative abundance profiles for nitrogen cycling bacteria (a), fungi (b), and archaea (c) from the 54‐plot AM to ECM gradient. This subset of phyla account for 0.37% of all sequencing reads. Each segment is the mean relative abundance and the error bars are standard error. The mycorrhizal category included three levels grouped into AM‐soil (plots ≥ 85% AM tree species), mixed‐soil (<85% AM and ECM tree species), and ECM‐soil (plots ≥ 85% ECM tree species). Sample size for ECM = 25, Mixed = 20, AM = 21. (d) Non‐metric multidimensional scaling plot for the distribution of N‐cycle taxa in relation to mycorrhizal type as well as ellipses (95%) for sampling sites

**Figure 4:**
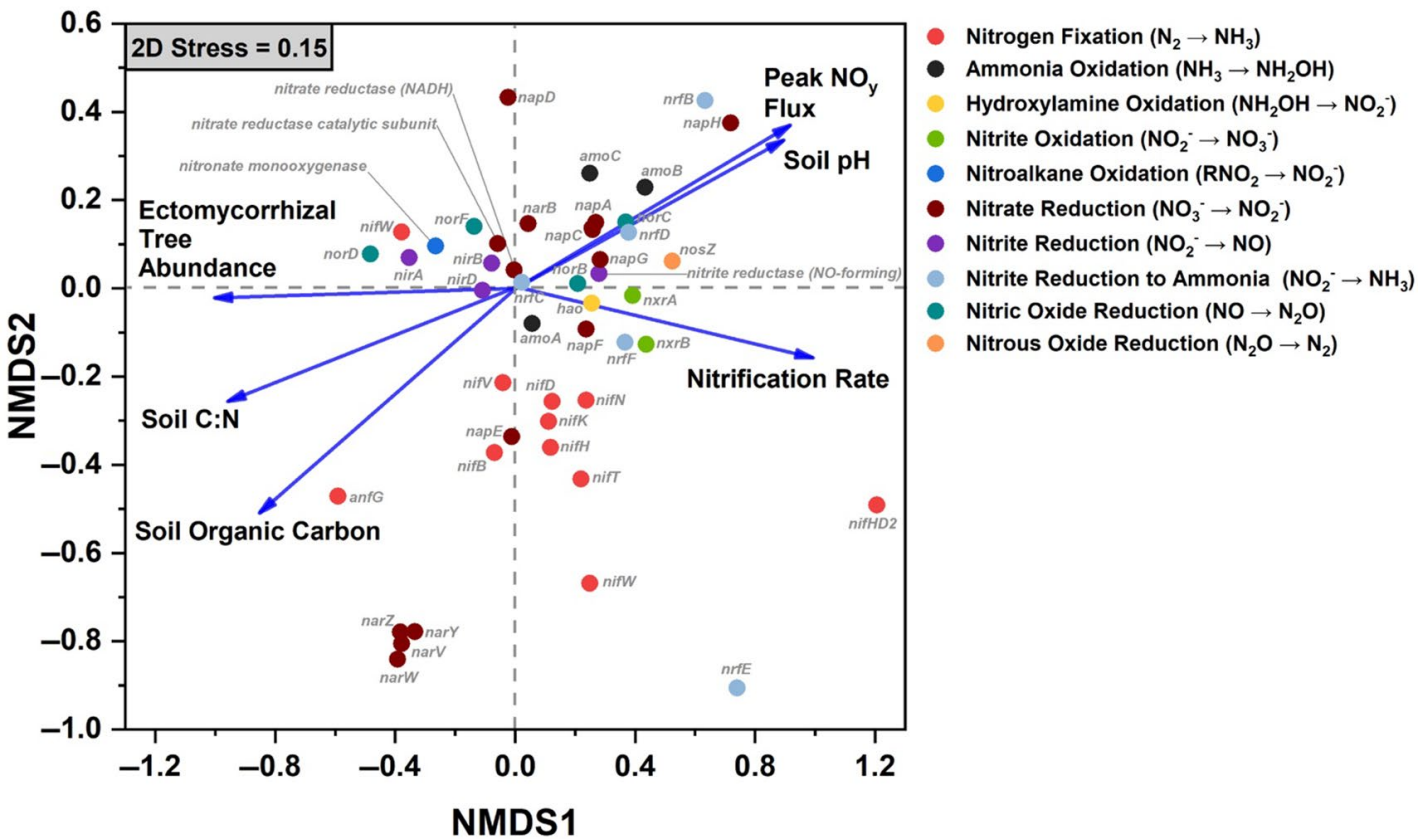
Non‐metric multidimensional scaling (NMDS) plot showing the distribution of nitrogen cycling genes (based on relative abundance values) and significant environmental properties as vectors (*p* < .05). Data for the NMDS are taken from the 54‐plot gradient

Within gradient plot soil, the N‐cycle community accounted for an average of 0.37% of all sequencing reads. Of the taxa with N‐cycle capability, Proteobacteria, Actinobacteria, and Acidobacteria were dominant, with the Proteobacteria accounting for 70 ± 6% (mean ± *SD*) of the total N‐cycle community across all soils (Figure 3a). Of the N‐cycling Proteobacteria, genera such as *Sorangium*, *Shewanella*, and *Ralstonia* were quite abundant in AM soil, and decreased in abundance as ECM tree abundance increased (Figure S1). In contrast, other dominant genera including *Mycobacterium* (Actinobacteria) and *Acidobacter* (Acidobacteria) increased significantly as ECM tree abundance increased (Figure S1). N‐cycle fungi and archaea accounted for less than one percent of the total N‐cycle community, and were influenced by sample location (Figure 3b,c). The N‐cycling Crenarcheota, were lower in the ECM soil (AM + Mixed versus ECM: *p* < .05). The overall structure of the N‐cycle community was significantly influenced by both dominant mycorrhizal classification of aboveground tree communities and site location (Figure 3d). N‐cycle community metrics were also influenced by tree assemblage with Simpson's index, Shannon's index, the number of N‐cycle taxa, and Pielou's evenness all decreasing as the abundance of ECM trees increased (Figure S2). Using NMDS, we assessed the relationship between the relative abundance of N‐cycle genes and soil physicochemical factors within the 54‐plot gradient (Figure 4). We found that peak NO*_y_* flux from soil microcosms and soil pH were linked to higher abundances of genes in the *amo*, *nap*, *nor*, and *nrf* operons. Net nitrification rate was linked closely to the positive NMDS axis 1, along with nitrification genes *amoA*, *hao*, and *nxrAB*. Ectomycorrhizal tree abundance was almost exclusively correlated to the negative NMDS axis 1, which also correlated with NIR and NOR genes. The two other significant vectors (soil C:N and soil organic carbon content) were not closely associated with any genes other than the nitrogen fixation gene, *anfG*.

**Figure 5:**
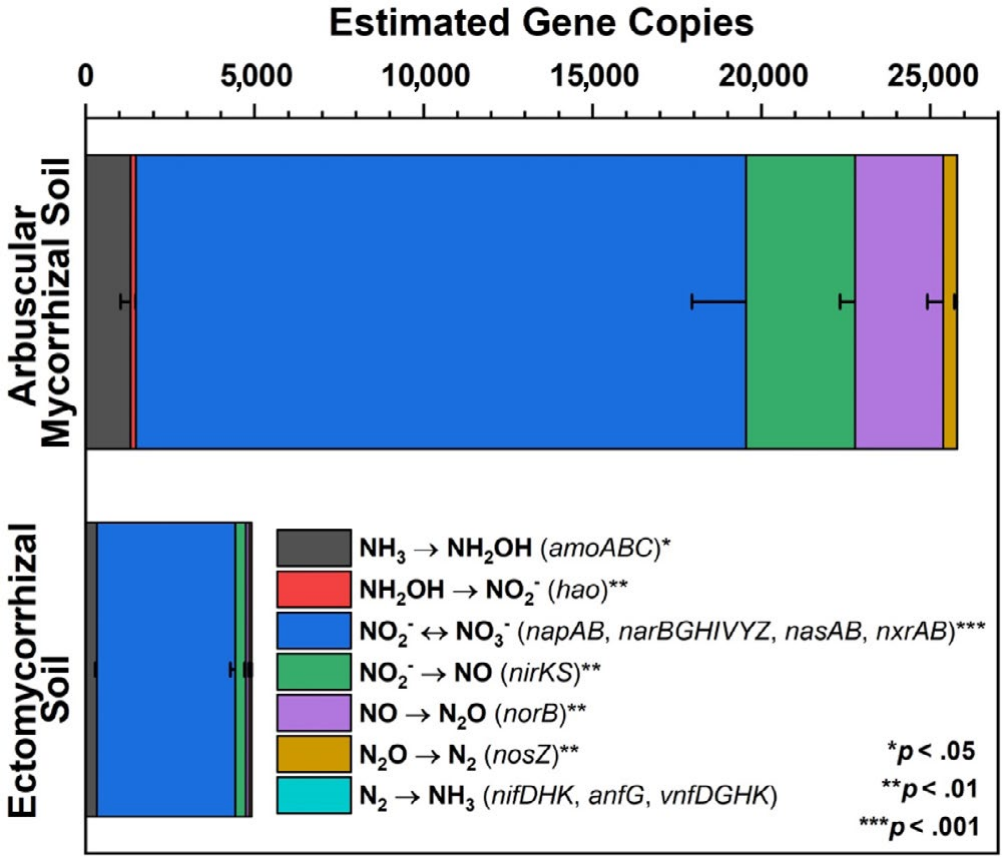
Estimated gene copies of key nitrogen cycle genes defined from metagenomic sequencing. Asterisks indicate level of significant difference between dominant mycorrhizal soil types taken from AM‐ and ECM‐dominated soil (Moores Creek). Estimated gene copy numbers were calculated as the number of genes multiplied by the average coverage of the contigs, on which these genes were predicted. Error bars indicate standard error of three replicates

**Figure 6:**
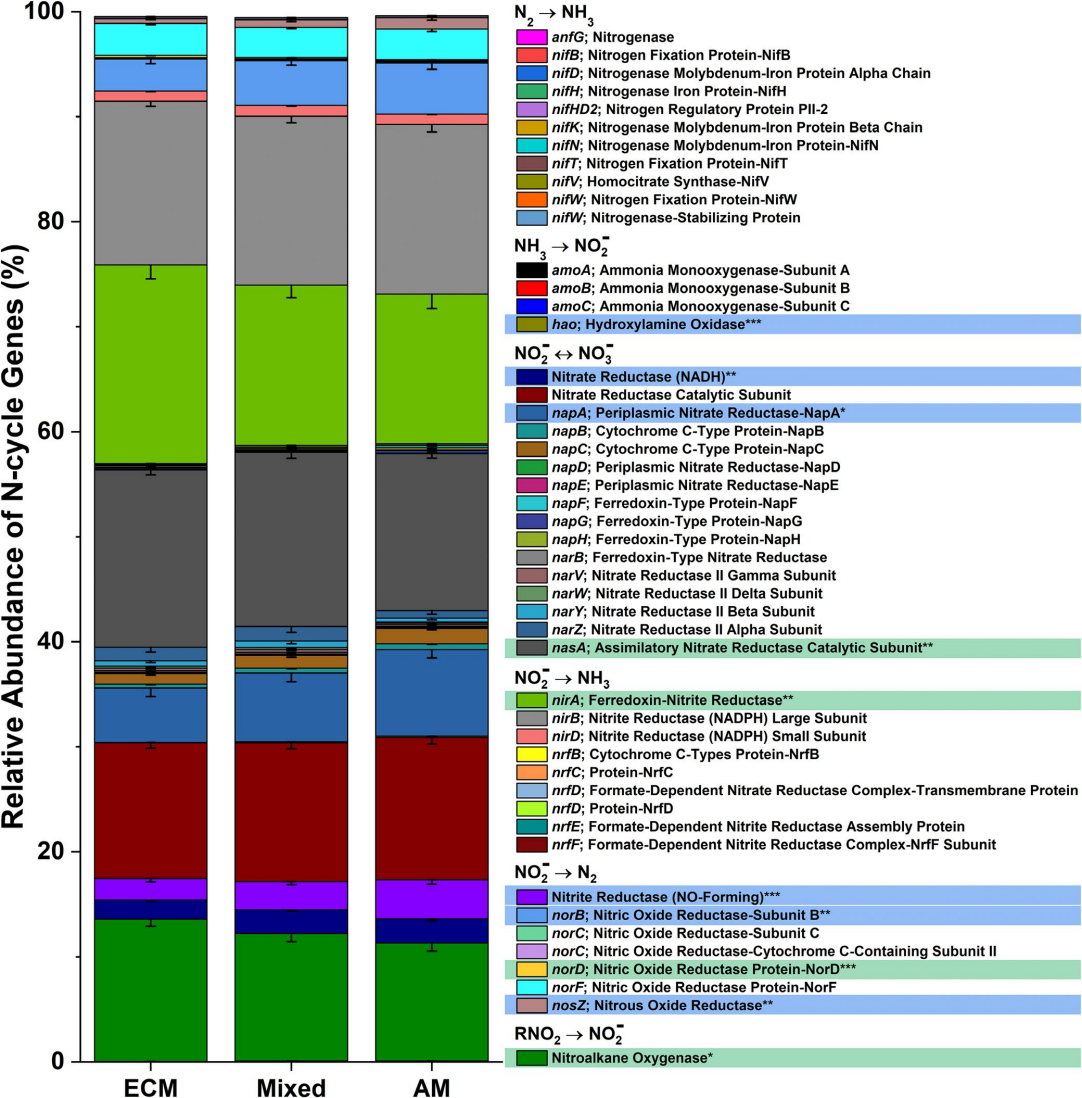
The relative abundance of nitrogen cycle genes across the 54‐plot gradient. The mycorrhizal category included three levels grouped into AM‐soil (plots ≥ 85% AM tree species), mixed‐soil (<85% AM and ECM tree species), and ECM‐soil (plots ≥ 85% ECM tree species). Sample size for ECM = 25, Mixed = 20, AM = 21. Genes highlighted blue are significantly more abundant in AM stands, while those highlighted in green are more abundant in ECM stands

Deep shotgun sequencing for Moores Creek soil showed AM soil possessed greater potential for all N‐cycle transformations except nitrogen fixation, which was relatively low in both AM and ECM soil (Figure 5). The highest estimated copy number was associated with nitrite/nitrate oxidation and reduction pathways, with *nasA* (assimilatory nitrate reductase catalytic subunit) accounting for 41% and 73% of all reported N‐cycle genes in AM‐ and ECM‐dominated soil, respectively (Table S1, Figure S3). Analysis of N‐cycle gene distribution for Moores Creek soil is consistent with the gradient plot soil, showing the relative proportion of genes associated with hydroxylamine oxidation, nitrite reduction, nitrate reduction, nitric oxide reduction, and nitrous oxide reduction increased from ECM to AM (Figure 6). Conversely, the relative abundance of N‐conservation genes associated with nitrite reduction (*nirA*), nitrate reduction (*nasA*), and nitroalkane oxidation increased in conjunction with increasing ECM tree abundance for gradient plot soils (Figure 6).

### 3.2 NO_y_ and N_2_O flux

**Figure 7:**
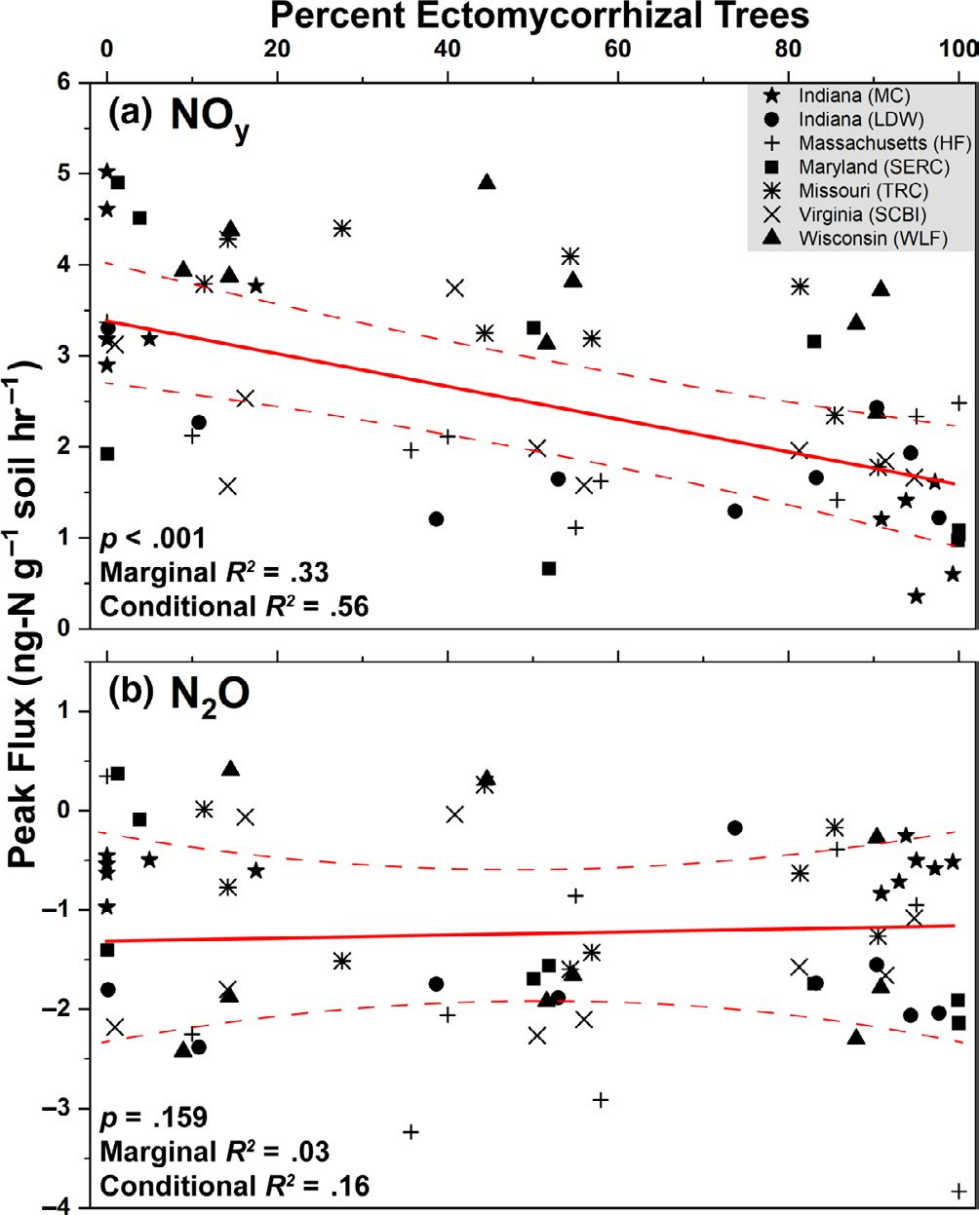
Results from a linear mixed effects model where peak NO*_y_* (a) and N_2_O (b) flux were the response variables and the percent of ectomycorrhizal trees was the independent variable. Data are combined from both sets of sites (Moores Creek and gradient plots). Site location was assessed as a random effect. The solid red line is the regression equation and dashed lines represent the 95% confidence intervals. The *p*‐values indicate the significance of the independent predictor (percent ectomycorrhizal trees) on peak fluxes of NO*_y_* and N_2_O. Peak fluxes were defined as the highest flux value over the course of the 48‐hr incubation. Marginal *R^2^* indicates the explained variability of gas fluxes solely on the effect of the independent predictor while the conditional *R^2^* accounts for variation of the independent predictor and the random effect (site location)

Across seven sites, peak NO_y_ flux decreased linearly from an average of 3.5 to 1.5 ng‐N g‐soil^−1^ hr^−1^ as the percentage of ECM trees increased from 0% to 100% (Figure 7a). ECM tree abundance accounted for 33% of variation in NO*_y_* flux (marginal *R^2^*), while ECM tree abundance combined with the random site factor accounted for 56% of NO*_y_* flux variability (conditional *R^2^*). Peak N_2_O flux ranged from 0.5 to –3 ng‐N g‐soil^−1^ hr^−1^, did not vary by site, and did not correlate to changes in ECM tree abundance (Figure 7b).

### 3.3 Oxic and anoxic microcosms

Fourteen‐day incubations of AM‐ and ECM‐dominated soil (Moores Creek soil) under differing incubation headspace atmospheres (e.g., oxic vs. anoxic) altered concentrations of aqueous oxidized nitrogen (Figure S4). Concentrations of ${\text{NO}}_{3}^{‐}$ were always greater in AM soil than those in ECM soil. For AM soil, oxic incubation led to an increase of 9.3 μg‐N g‐soil^−1^ (+98%) relative to field‐levels (*p* < .001) while anoxic incubation resulted in a decrease of 4.0 μg‐N g‐soil^‐1^ (−58%; *p* < .1). In ECM soil, initial levels of ${\text{NO}}_{3}^{‐}$ were 8.6 μg‐N g‐soil^−1^ lower than the AM soil. Oxic and anoxic incubations of ECM soil led to a decrease of 0.8 and 0.9 μg‐N g‐soil^−1^, which represents a −89% and −95% change, respectively (*p* < .001).

**Figure 8:**
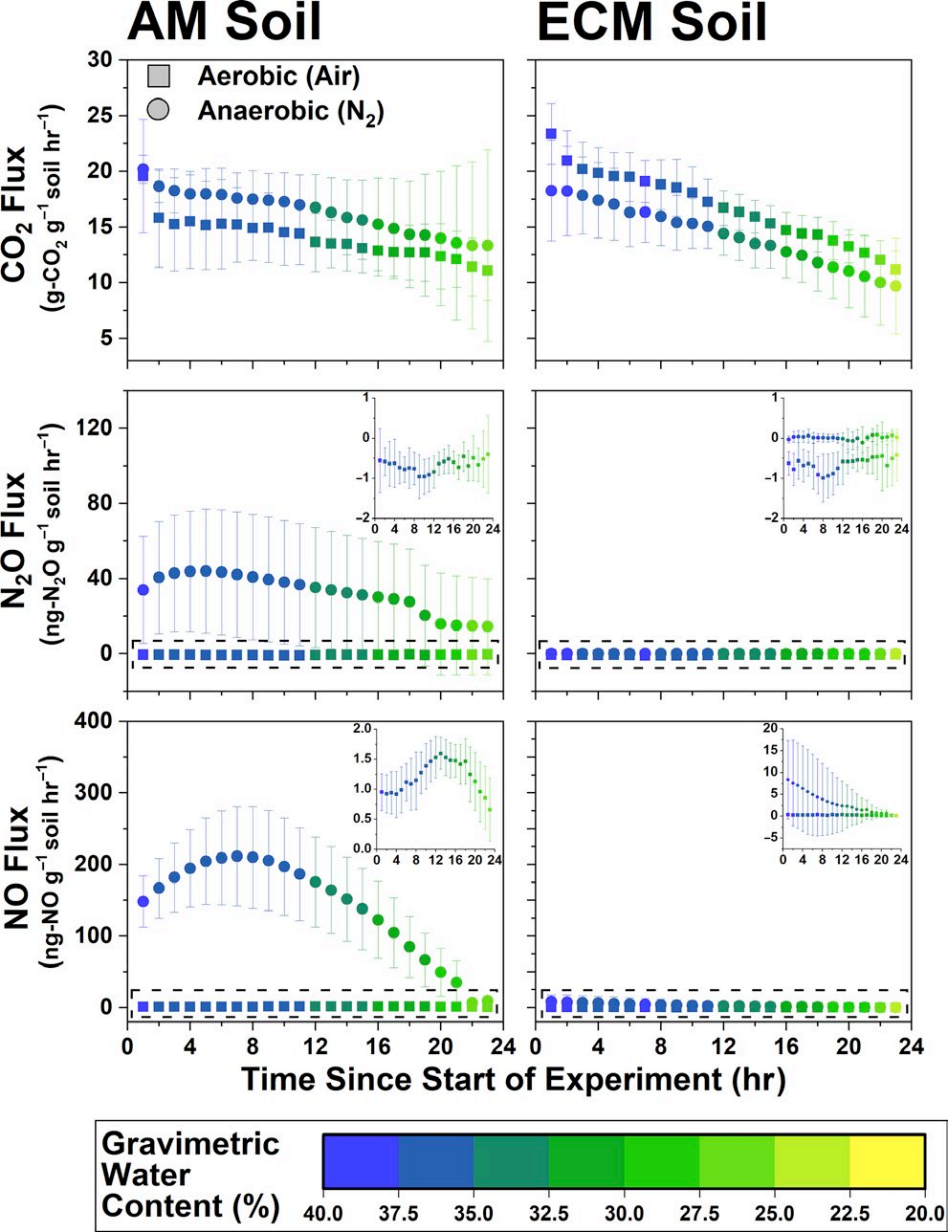
Gas fluxes (CO_2_, N_2_O, and NO) for 24 hr incubations under oxic (ultra‐pure air: 20% O_2_, 80% N_2_) and anoxic (>99% N_2_) conditions. Inset for fluxes shows finer resolution of data within dashed boxes. Soil used in this experiment is from AM‐ and ECM‐dominated plots at Moores Creek. For each data series, *N* = 8.

Within Moores Creek samples, gas fluxes from both AM and ECM soil were heavily influenced by water content, and generally decreased as soil dried (Figure 8). CO_2_ flux (microbial respiration) did not vary in response to incubation atmosphere conditions nor mycorrhizal soil type, which is consistent with what was observed when peak CO_2_ flux from the AM to ECM gradient plots was analyzed in response to ECM abundance (Figure S5). For N_2_O and NO, higher values under anoxic conditions were observed for the AM soil (NO: 145.4 ± 75.5 ng‐N g‐soil^−1^ hr^−1^; N_2_O: 31.8 ± 30.2 ng‐N g‐soil^−1^ hr^−1^). ECM soil fluxes of NO_y_ and N_2_O were extremely low and differences between oxic and anoxic conditions were negligible (Figure 8). NO flux in AM soil peaked at 13 and 7 hr under oxic and anoxic conditions, respectively. In ECM soil, flux decreased linearly from 0.32 to 0.12 ng‐N_NO_ g‐soil^−1^ hr^−1^ under oxic conditions and from 8.35 to <0.1 ng‐N_NO_ g‐soil^−1^ hr^−1^ under anoxic conditions. Only AM soil produced a definitive peak N_2_O flux, which occurred at 5 hr under anoxic conditions (44.0 ng‐N_N2O_ g‐soil^−1^ hr^−1^).

Using RT‐qPCR, we determined that transcript abundance of AOA and AOB *amoA* was higher under oxic conditions for Moores Creek soil and tended to decrease with time (Figure S6). For AOA *amoA*, the average number of transcripts was 72% higher in AM soil relative to ECM soil (*p* < .001), while AOB *amoA* transcript numbers were 177% higher in AM soil (*p* < .001). Regardless of soil type or oxygen availability, denitrification transcript abundance followed *nosZ* (Clade I) > *nirK* > *nirS*. For AM soil, denitrification transcripts were higher under anoxic conditions; however, in ECM soil, there was no difference in transcript copies for the key N‐cycle genes between oxic and anoxic incubations. Incubation time (i.e., 8 and 24 hr) influenced *nirK* and *nosZ* (Clade I) where 8 hr < 24 hr, but not *nirS*. Many of the transcript levels for ECM soil were below detection limits.

### 3.4 Mixed effects models—factors responsible for NO_y_ and N_2_O flux

**Figure 9:**
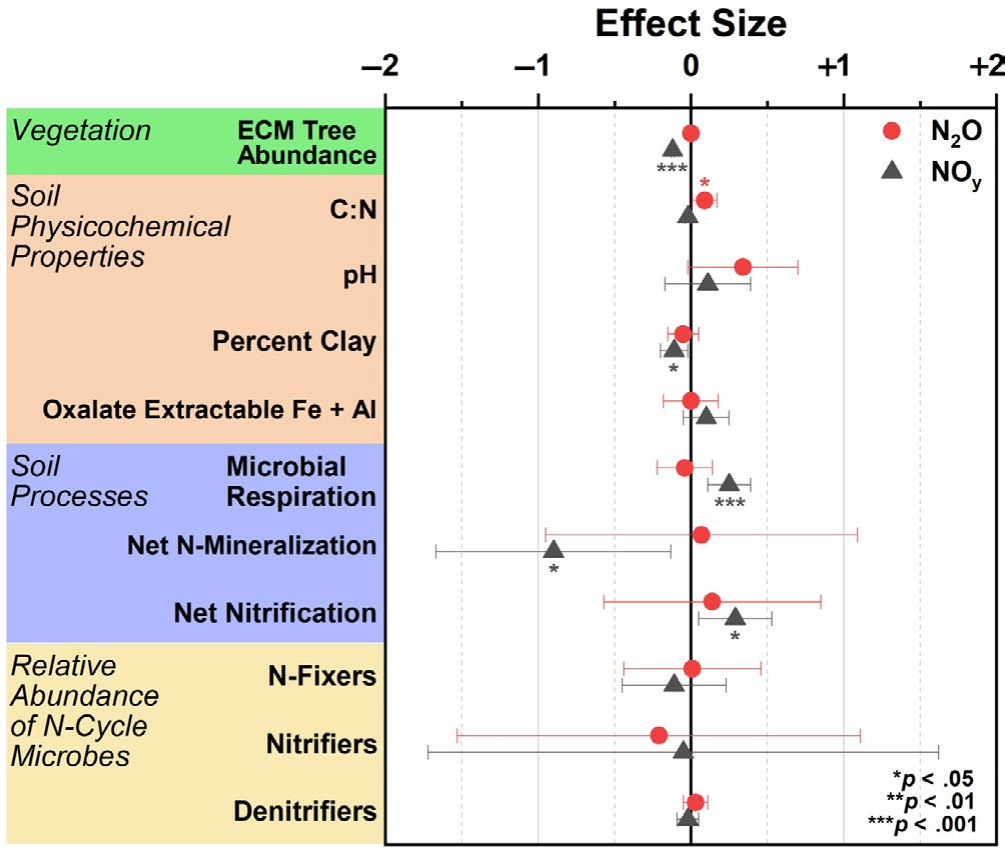
Effect size from a linear mixed effects model where nitrogen oxide flux (N_2_O or NO*_y_*) was the response variable and edaphic parameters including vegetation composition, soil physicochemical properties, soil processes, and the relative abundance of nitrogen cycling microbial guilds were fixed predictive variables. Data were used from the gradient plots. Geographic location was used as a random effect. *R^2^* for NO*_y_* was 0.38/0.80 (marginal *R^2^*/conditional *R^2^*) and 0.18/0.39 for N_2_O. Model intercepts: NO*_y_* = 3.43 ± 5.21; N_2_O = −5.45 ± 6.32

In gradient soil, fixed effects (ECM tree abundance, C:N, pH, percent clay, oxalate extractable iron and aluminum accounted, microbial respiration, net N mineralization, net nitrification, and the relative abundance of N fixers, nitrifiers, and denitrifiers) accounted for 18% and 38% of variation (marginal *R^2^*) for peak N_2_O and NO_y_ flux, respectively (Figure 9). This level of variance was significant for NO_y_ but not N_2_O flux. Conditional *R^2^* was 39% (N_2_O) and 80% (NO*_y_*), indicating that the random factor (geographic location) plays a large role in nitrogen oxide flux variation across a spatial gradient. For N_2_O flux, soil C:N was the only factor with a significant effect size, while variables such as ECM tree abundance, percent clay, microbial respiration, net nitrogen mineralization, and net nitrification were significantly predictive of NO_y_ flux (Figure 9).

## 4 DISCUSSION

### 4.1 Metagenomic analyses reveal greater potential for N‐cycle activity in AM soil

The higher abundance of N‐cycle genes such as *amoABC*, *hao*, and *nxrAB* in AM‐dominated soils are in agreement with previous studies showing that under oxic conditions, AM soils have greater rates of net nitrification (Midgley & Phillips, 2016; Mushinski et al., 2019; Phillips et al., 2013), which likely explains high rates of oxic NO_y_ flux shown in AM soils (Figure 7). Estimated gene copy numbers for *norB* and *nosZ*, which code for nitric oxide reductase and nitrous oxide reductase, respectively, were both higher in AM‐dominated soil (Figure 5) and the relative abundance of these same genes were in greater proportion in AM soil along the 54‐plot gradient (Figure 6). This indicates that under reducing conditions, AM soil has the genomic potential for volatile nitrogen oxide flux to be much higher relative to ECM soil, which was verified by flux measurements conducted under anoxic conditions (Figure 8). *NasA* was by far the most abundant N‐cycle gene in Moores Creek soil and was the third most abundant N‐cycle gene in the gradient plots. *NasA* was also significantly more abundant in all ECM soils tested (Moores Creek and gradient soils). This gene codes for an assimilatory nitrate reductase catalytic subunit which reduces ${\text{NO}}_{3}^{‐}$ to ${\text{NO}}_{2}^{‐}$ and is associated with microbial uptake of ${\text{NO}}_{3}^{‐}$. The high estimated *nasA* copy numbers in Moores Creek soil indicate the importance of microbial uptake and conservation of oxidized N in these forests systems. Furthermore, the high proportion of *nasA* in ECM soil indicates high uptake and utilization of any free ${\text{NO}}_{3}^{‐}$, which may partially explain low concentrations of ${\text{NO}}_{3}^{‐}$ in ECM soil. While AM soils also have high relative abundance of *nasA,* ${\text{NO}}_{3}^{‐}$ uptake is likely supplemented by high rates of nitrification and ammonification, resulting in a very fast N cycle in AM soil.

AM‐induced microbial stimulation coupled with higher litter quality and fast turnover rates may explain why genes associated with heterotrophic microbes such as denitrification are significantly higher in the AM soil (Figure 5), leading to higher fluxes of nitrogen oxide, specifically NO*_y_* (Figures 7, 8). AM soils throughout these sites (Craig et al., 2018; Midgley et al., 2015) and elsewhere (Craig et al., 2018; Keller & Phillips, 2019; Lin et al., 2017) have leaf litter layers of low lignin:N ratios (i.e., higher chemical quality). Given the well‐established relationship between fast cycling litters and N availability in forests (Scott & Binkley, 1997), and the relationship between inorganic N pools and nitrification fluxes (Persson et al., 2000), we expect microbes responsible for accelerating the N cycle to be more common in AM soils. However, differences in the physiology and metabolism of mycorrhizal fungi may also contribute to the observed differences in microbial community composition between AM and ECM soils. Although ECM fungi possess hydrolytic capabilities that can result in the slow liberation of organic C and N from complex SOM (Kohler et al., 2015; Lindahl & Tunlid, 2015), AM fungi can release labile carbon into the hyphosphere, leading to greater stimulation of heterotrophic microbes (Herman et al., 2012; Kuzyakov, 2010; Paterson et al., 2016; Talbot et al., 2008), and possibly greater litter decay rates (Bunn et al., 2019). ECM fungi produce significant amounts of mycelium which may function as a large sink of inorganic N (Högberg & Högberg, 2002), possibly shaping the free‐living N‐cycle community in ECM soil toward more N conservation rather than processes leading to N loss such as nitrification and denitrification. Quantification of AM and ECM fungi within these soils would better elucidate the importance of these potential mechanisms.

It is unknown how differences in AM and ECM microbial communities influence precursory processes to nitrification, such as ammonification. Pools of ammonium were similar in AM‐ and ECM‐dominated soil but rates of ammonium accumulation were significantly higher in ECM soil (Figure S7), indicating a potential bottleneck at the nitrification step. This may be a function of the higher acidity observed in ECM soil, which directly influences rates of nitrification through the protonation of ammonia to ammonium (NH_4_^+^p*K*_a_ = 9.3); this renders the substrate less available for nitrification and other downstream processes, inevitably selecting for lower abundances of ammonia oxidizers, as seen previously (Mushinski et al., 2019). Soil pH (which positively correlates with ECM tree abundance) can also influence N cycling by modifying microbial diversity. Less acidic AM soils support greater microbial diversity (Fierer & Jackson, 2006) and higher N‐cycle potential (Figure S2; Figure 5)—heterotrophic microbes involved in mineralization are likely affected by soil pH in the same manner. Further inhibition may be mediated by the presence of litter‐derived polyphenolic compounds, which tend to be more abundant in ECM soil (Subbarao et al., 2013). This nitrification bottleneck hypothesis is further supported by a supplemental experiment showing that when ECM soil is supplemented with inorganic *N* (nitrite) under anoxic conditions, increases in N_2_O production and denitrification transcripts are observed (Figure S8). This indicates that ECM soil is inherently able to produce nitrogen oxides, but the process is repressed due to a lack of available substrate, likely as a result of the inhibition of nitrification.

### 4.2 Oxygen availability alters N‐cycle rates and microbial activity in AM soil

Variations in oxygen availability can drive dramatic changes in nitrogen oxide production by stimulating different groups of N‐cycle microbes. We hypothesized that anoxic conditions would lead to greater emissions of nitrogen oxides for both AM and ECM soil, with AM soil producing significantly greater N‐gas fluxes under anoxic conditions. Our results partially support this hypothesis; there was no significant response from ECM soil to anoxic conditions. Consistent with our hypothesis, AM soil produced more NO and N_2_O relative to ECM soil (Figure 8)—likely a direct result of higher oxidized N availability and greater potential for denitrification. This observation agrees with a field‐based study which showed that under poorly drained (partially anoxic) conditions, AM (maple) stands produced three to four times more N_2_O relative to ECM (beech) stands (Ullah & Moore, 2011). The difference between anoxic and oxic NO and N_2_O flux in AM soil was extremely large, relative to ECM soil. NO flux increased by roughly 144 ng‐N g‐soil^−1^ hr^−1^ and N_2_O flux increased by 32 ng‐N g‐soil^−1^ hr^−1^ under anoxic conditions in AM soil, which was more than the 3 and 0.8 ng‐N g‐soil^−1^ hr^−1^ increases in ECM soil. The lower flux levels for N_2_O may be a result of greater N_2_O to N_2_ conversion, a prediction that agrees with *nosZ* having high gene expression. Furthermore, at its highest flux value (i.e., at hour 8 of the incubation), the amount of N being lost from AM soil accounted for roughly 2% of the entire pool of NO_3_^−^. Soil transcript abundance of key N‐cycle genes confirmed that observed fluxes were the result of biological processes where, under oxic conditions, nitrification transcripts (AOA and AOB *amoA*) were more abundant than denitrification transcripts (*nirS*, *nirK*, *nosZ*), and vice versa under anoxic conditions (Figure S6). It is also worth noting that AM forest soil may be inherently more anoxic due to AM fungi promoting soil aggregation through the release of labile organic compounds (Wright et al., 1999).

The large discrepancy between AM and ECM soil nitrogen oxide flux has substantial implications for future forest dynamics. Even currently, individual AM trees in ECM‐dominated forests likely produce soil hotspots of nitrogen oxide flux regardless of soil oxygen condition. Upland forest soils have generally been shown to be sources of N_2_O and NO; however, the magnitude of this source depends on vegetation composition, geographic location, and previous land‐use history (Butterbach‐Bahl et al., 2013; Chapuis‐Lardy et al., 2006; Pilegaard, 2013). Systems with highly conservative N‐cycle dynamics, as seen in oak‐hickory‐beech (ECM) forests throughout the eastern United States, are presumed to contribute much less relative to adjacent agricultural systems; however, fast N‐cycling AM forests have not been considered. It is likely that these forests do not naturally receive enough N inputs to compare to fluxes measured from major US crops such as corn (Decock, 2014) (0 to 30 kg‐N_N__2O_ ha^–1^ year^−1^) and switchgrass (Ruan et al., 2016) (0 to 6 kg‐N_N__2O_ ha^–1^ year^−1^), which typically receive large amounts of N fertilizer (up to 200 kg‐N_fertilizer_ ha^–1^ year^−1^). For reference, temperate forests of the eastern United States are estimated to receive 10–15 kg‐N/ha^–1^ year^−1^ via atmospheric deposition (Schwede et al., 2018). However, our observation that anoxic soil conditions stimulate microbial denitrification and large increases in N_2_O and NO flux for AM soil is noteworthy, especially in light of the increasing abundance of AM trees across much of the eastern United States (Jo et al., 2019). While it has typically been assumed that well‐drained upland soils are completely oxic, 2%–9% of the pore volume can be anoxic, even when bulk soil O_2_ concentrations are high (Keiluweit et al., 2018). This indicates that in situ N gas fluxes from forest soils may be higher than what is currently assumed and should be compared to adjacent natural and managed ecosystems.

### 4.3 Multiple factors influence the production of volatile nitrogen oxides in forest soil

Results from Figure 9 illustrate that reactive NO_y_ fluxes are best explained primarily by the relative abundance of ECM trees species and to a lesser degree, the percentage of clay and soil processes such as microbial respiration, net N mineralization, and net nitrification. Except for net nitrification, none of these factors were included in a previous study (Mushinski et al., 2019). The significant influences of ECM on NO*_y_* flux is likely due to combined effects of ECM trees in lowering soil pH due to acidifying processes associated with litter decomposition. This in turn lowers the abundance of available ammonia, nitrifiers, and nitrification rates, which are all directly related to NO*_y_* flux. It was surprising that soil clay content was a significant factor in this model due to its lack of correlation to ECM tree abundance (Figure S5); however, clay content is likely important in modulating oxic and anoxic conditions, soil porosity, and soil reactivity which may influence NO*_y_* production (Kebede et al., 2016; Maharjan & Venterea, 2014). Furthermore, the amount of variance explained increased from 23% in the initial model (Mushinski et al., 2019) to 39% for fixed effects only and 80% for fixed effects plus the influence of geographic location (mixed model, this paper). Note that the relative abundance of N‐cycle microbes was not found to be a significant predictive variable for oxic NO*_y_* fluxes. Mushinski et al. (2019) identified nitrifying microbes (AOA and AOB) as contributing 60%–70% to oxic NO*_y_* flux from inhibitor assays; however, this was based on activity and not functional potential as shown here. RNA‐based methods will likely provide better resolution on the contribution of specific microbial guilds. Considering the large flux differences in response to oxygen availability, similar analyses should be carried out to determine the factors responsible for anoxic flux of nitrogen oxides in these systems. The varied responses of N_2_O and NO*_y_* in these experiments is difficult to interpret—considering they are both products of nitrification and denitrification. However, it may stem from multiple factors including unknown abiotic chemistry, an extremely efficient conversion of N_2_O to N_2_, or simply a function of the microcosms used in this study—it remains to be seen whether the microcosm chambers yield the same results as in situ chamber flux measurements. Although, studies in grasslands suggest good agreement between the two methods (van Dijk et al., 2002).

### 4.4 Synthesis

Changes in global climate and other disruptions such as atmospheric deposition of anthropogenic N are inducing shifts in the relative abundance of AM and ECM trees in temperate forests (Jo et al., 2019; Steidinger et al., 2019), with unknown consequences for biogeochemical cycling in these systems. Our study illustrates that the global change‐induced vegetation shifts currently being observed throughout the eastern United States (Jo et al., 2019) may significantly alter the soil bacterial and archaeal N‐cycling communities and have significant effects on atmospheric composition, especially in the wake of climate change. We hypothesized that forests with more “open” N cycles (e.g., AM‐dominated stands) contain microbial communities with greater numbers of N‐cycling taxa and genes that are specifically related to the production of volatile nitrogen oxides. Using forests throughout the eastern United States (Figure 2), we find support for this hypothesis: AM‐dominated soils had microbial communities with a greater relative abundance of N‐cycling genes. This in turn leads to greater metagenomic potential for ammonia oxidation, hydroxylamine oxidation, nitrite oxidation/reduction, nitrate reduction, nitric oxide reduction, and nitrous oxide reduction (Figure 5). Additionally, we observed mycorrhizal‐based differences in the N‐cycling bacterial consortia (Figure 3a–c), the structure of the N‐cycle microbial community (Figure 3d) as well as a different proportional profile of N‐cycle taxa and genes based on mycorrhizal association (Figure 6; Figure S3). The spatial variability of nitrogen oxide fluxes was also investigated. We found NO_y_ fluxes to be highest in AM soils across the eastern United States (Figure 7) and strongly correlated to the relative abundance of genes associated with nitrification and denitrification (Figure 4); however, the magnitude of flux was dependent on geographic location (mixed effects model). We also found support for our hypothesis that anoxic conditions lead to equivalent or greater emissions of nitrogen oxides for both AM and ECM soil, with AM soil producing more N_2_O and NO*_y_* under anoxic conditions (Figure 8)—likely a direct result of higher N availability and greater microbial potential for denitrification. Furthermore, the low levels of inorganic nitrogen in ECM soil may serve as a bottleneck to nitrogen oxide production (Figure S4), which was supported by a positive N_2_O response following addition of nitrite (Figure S8). Collectively, our results suggest that shifts in forest composition may have profound consequences for microbial communities involved in N cycling and the tendency of forest soil to modulate volatile nitrogen oxides. Furthermore, this study indicates that proliferation of AM species into ECM‐dominated ecosystems throughout temperate forest regions may represent a major change in the soil N‐cycle microbiome, possibly leading to ecosystem‐scale altering of soil N‐cycle process rates and high loss of soil N through volatilization, regardless of soil oxygen conditions.

## CONFLICT OF INTEREST

The authors claim no competing interests for this work.

## AUTHOR CONTRIBUTIONS

R.M.M. led the work; R.M.M., Z.C.P., J.D.R., and R.P.P. designed research; R.M.M., Z.C.P., and M.E.C. performed research; R.M.M., Z.C.P., J.D.R., M.E.C., S.E.P., J.R.W., and R.P.P. contributed new reagents/analytic tools; R.M.M., Z.C.P., J.D.R., M.E.C., D.B.R., and R.P.P. analyzed data; and R.M.M., Z.C.P., J.D.R., and R.P.P. wrote the paper.

## Supporting information

Supplementary Material

## Data Availability

The data that support the findings of this study are available from the corresponding author upon reasonable request.
