# Supplementary material for: Nitrogen cycling microbiomes are structured by plant mycorrhizal associations with consequences for nitrogen oxide fluxes in forests
Source: Glob Chang Biol. 2020 Dec 15;27(5):1068–82. doi: 10.1111/gcb.15439 (PMC7898693; doi:10.1111/gcb.15439)

Title: Nitrogen cycling microbiomes are structured by plant mycorrhizal associations with consequences for nitrogen oxide fluxes in forests

Running Title: Nitrogen cycle microbiomes in forest soils

Authors: Ryan M. Mushinski[1,2]*, Zachary C. Payne[2,3], Jonathan D. Raff[2,3], Matthew E. Craig[4,5], Sally E. Pusede[6], Douglas B. Rusch[7], Jeffrey R. White[2,8], Richard P. Phillips[4]

Author Affiliations:
[1] School of Life Sciences, University of Warwick, Coventry, UK
[2] School of Public and Environmental Affairs, Indiana University, Bloomington, IN, USA
[3] Department of Chemistry, Indiana University, Bloomington, IN, USA
[4] Department of Biology, Indiana University, Bloomington, IN, USA
[5] Environmental Sciences Division and Climate Change Science Institute, Oak Ridge National Laboratory, Oak Ridge, TN, USA
[6] Department of Environmental Sciences, University of Virginia, Charlottesville, VA, USA
[7] Center for Genomics and Bioinformatics, Indiana University, Bloomington, IN, USA
[8] Department of Earth and Atmospheric Sciences, Indiana University, Bloomington, IN, USA

*Corresponding author: Ryan M. Mushinski

Mailing Address:
Gibbet Hill Campus
University of Warwick
Coventry CV4 7AL
United Kingdom

Corresponding Author Contact Information:
Email: Ryan.Mushinski@warwick.ac.uk
Phone: +44 (0)24 76 575050

Table of Contents

Page Number

Supplementary Tables

Table S1: Nitrogen cycle gene abundances S3

Table S2: Primer and amplification information for N-cycle qPCR S4

Table S3: Distribution of N-cycle phyla S5

Supplementary Figures

Figure S1: Regression of N-cycle genera against ECM tree abundance S6

Figure S2: Regression of N-cycle community metrics against ECM tree abundance S7

Figure S3: Proportional profile of N-cycle genes (Moores Creek) S8

Figure S4: Soil nitrite and nitrate concentrations S9

Figure S5: Soil physicochemical properties S10

Figure S6: Transcript copies of key nitrogen cycle genes in AM and ECM soil S11

Figure S7: Soil ammonium concentrations and ammonification rates S12

Figure S8: Time series showing denitrification activity in ECM spiked with nitrite S13

Table S1: Nitrogen cycle gene abundances and statistical differences between arbuscular mycorrhizal (AM) and ectomycorrhizal soil (ECM) dominated soil at Moores Creek. Estimated gene copy numbers were calculated as the number of genes multiplied by the average coverage of the contigs, on which these genes were predicted.

Process	Gene of Interest	KEGG Ortholog	***Estimated Gene Copies*** AM (*n*=3)	ECM (*n*=3)	Fisher's Exact Test
$NH_3 \rightarrow NH_2OH$	*amoA*	KO:K10944	337 ± 155	92 ± 28	p<0.001
	amoB	KO:K10945	350 ± 109	109 ± 21	p<0.001
	amoC	KO:K10946	633 ± 253	126 ± 37	p<0.001
$NH_2OH \rightarrow NO_2^-$	*hao*	KO:K10535	170 ± 38	0 ± 0	p<0.001
$NO_2^- \leftrightarrow NO_3^-$	*narG, narZ, nxrA*	KO:K00370	2403 ± 859	155 ± 41	p<0.001
	narH, narY, nxrB	KO:K00371	1146 ± 344	86 ± 22	p<0.001
	narI, narV	KO:K00374	226 ± 40	98 ± 37	p<0.001
	nasA	KO:K00372	10599 ± 1362	3601 ± 268	p<0.001
	nasB	KO:K00360	95 ± 25	10 ± 5	p<0.001
	napA	KO:K02567	2307 ± 298	55 ± 25	p<0.001
	napB	KO:K02568	437 ± 61	18 ± 6	p<0.001
	narB	KO:K00367	837 ± 318	76 ± 22	p<0.001
$NO_2^- \rightarrow NO$	*nirS*	KO:K15864	74 ± 25	43 ± 17	Not Significant
	nirK	KO:K00368	3221 ± 749	307 ± 63	p<0.001
$NO \rightarrow N_2O$	*norB*	KO:K04561	2610 ± 800	95 ± 22	p<0.001
	norC	KO:K02305	212 ± 45	7 ± 7	p<0.001
$N_2O \rightarrow N_2$	*nosZ*	KO:K00376	408 ± 114	84 ± 23	p<0.001
$N_2 \rightarrow NH_3$	*nifH*	KO:K02588	10 ± 8	1 ± 1	Not Significant

Table S2: Primer and amplification information for genes used in qPCR experiments.

Gene	Forward Primer (5'-3')	Reverse Primer (5'-3')	Annealing T (°C)	qPCR Result
AOA-*amoA*	Arch amoA-1F (STAATGGTCTGGCTTAGACG)[a]	Arch amoA-2R (GCGGCCATCCATCTGTATGT)[a]	55	+
AOB-*amoA*	amoA-1F (GGGGTTTCTACTGGTGGT)[b]	amoA-2R (CCCCTCKGSAAAGCCTTCTTC)[b]	55	+
nirS	nirS1F (CCTAYTGGCCGGCRCART)[c]	nirS3R (GCCGCCGTCRTGVAGGAA)[d]	56	+
nirK	F560-589 (GGGCATGAACGGCGCGCTCATGGTGCTGCC)[e]	R906-935 (CGGGTTGGCGAACTTGCCGGTGGTCCAGAC)[d]	55	+
norB	qnorB2F (GGNCAYCARGGNTAYGA)[f]	qnorB5R (ACCCANAGRTGNACNACCCACCA)[f]	55	BD
	cnorB1F (GARTTYCTNGARCARCC)[f]	cnorB7R (TGNCCRTGNGCNGCNGT)[f]	55	BD
nosZ	nosZ-F (CGYTGTTCMTCGACAGCCAG)[g]	nosZ-R (CATGTGCAGNGCRTGGCAGAA)[g]	56	+

[a] Francis, C. et al. 2005. Proceedings of the National Academy of Sciences USA 102, 14683-14688.

[b] Rotthauwe, J.H. et al. 1997. Applied and Environmental Microbiology 63, 4704-4712.

[c] Braker, G. et al. 1998. Applied and Environmental Microbiology 64, 3769-3775.

[d] Levy-Booth, D.J. and Winder, R.S. 2010. Applied and Environmental Microbiology 76, 7116-7125.

[e] Chénier, M.R. et al. 2003. Applied and Environmental Microbiology 69, 5170-5177.

[f] Braker, G. and Tiedje J. 2003. Applied and Environmental Microbiology 69, 3476-3483.

[g] Rösch, C. et al. 2002. Applied and Environmental Microbiology 68, 3818-3829.

Table S3: Nitrogen cycling phyla from the 54-plot gradient. Text with the parenthesis indicate the mycorrhizal type or site where the relative abundance was highest. For site, the locations are in order of highest abundance to lowest.

Kingdom	Phylum	N-Cycle Community Percentage *Mean ± S.E.*	Mycorrhizal Type (M)	Site (S) *F-Ratio*	M × S
Archaea	Crenarchaeota	< 0.01	2.66	1.03	2.31* (AM × SERC)
	Euryarchaeota	0.14 ± 0.02	0.48	5.81*** (TRC, SCBI, LDW, WLF, SERC, HF)	1.06
	Thaumarchaeota	0.01 ± 0.01	0.37	1.32	0.37
Bacteria	Acidobacteria	5.51 ± 0.32	3.05	8.23*** (HF, LDW, SERC, WLF, TRC, SCBI)	2.03
	Actinobacteria	18.47 ± 0.81	4.34* (ECM)	3.09* (LDW, HF, SCBI, SERC, WLF, TRC)	1.41
	Aquificae	0.15 ± 0.01	3.86* (AM)	1.59	0.75
	Bacteroidetes	1.11 ± 0.09	1.88	4.58** (TRC, SERC, WLF, SCBI, LDW, HF)	2.07
	Chlamydiae	0.09 ± 0.01	3.91* (Mixed)	1.35	1.24
	Chlorobi	0.09 ± 0.01	0.02	1.31	0.71
	Chloroflexi	0.20 ± 0.02	5.72** (AM)	9.17*** (TRC, WLF, SERC, SCBI, LDW, HF)	1.75
	Chrysiogenetes	< 0.01	0.16	0.75	0.91
	Cyanobacteria	0.59 ± 0.02	0.92	1.45	0.68
	Deferribacteres	0.03 ± 0.01	1.31	1.28	1.28
	Deinococcus-Thermus	0.24 ± 0.01	4.81* (AM)	4.63** (TRC, LDW, WLF, SCBI, SERC, HF)	0.91
	Elusimicrobia	< 0.01	0.21	0.81	1.61
	Firmicutes	1.09 ± 0.06	0.51	4.25** (TRC, HF, SERC, LDW, WLF, SCBI)	2.06
	Fusobacteria	< 0.01	1.02	0.93	1.01
	Gemmatimonadetes	0.16 ± 0.02	5.17* (AM)	1.24	0.69
	Nitrospirae	0.12 ± 0.03	4.29* (AM)	8.76***(TRC, LDW, WLF, SCBI, SERC, HF)	1.82
	Planctomycetes	0.92 ± 0.04	0.58	4.29** (LDW, TRC, SCBI, WLF, SERC, HF)	0.69
	Proteobacteria	69.66 ± 0.85	2.98	2.69* (WLF, TRC, SCBI, SERC, LDW, HF)	1.36
	Spirochaetes	0.10 ± 0.01	4.11* (AM)	7.68*** (TRC, LDW, SCBI, SERC, WLF, HF)	1.11
	Unclassified Bacteria	0.16 ± 0.02	0.55	2.09	1.56
	Verrucomicrobia	0.93 ± 0.05	3.09	4.53** (TRC, LDW, WLF, SERC, SCBI, HF)	1.21
Fungi	Ascomycota	0.20 ± 0.02	1.07	7.69*** (LDW, TRC, SCBI, SERC, WLF, HF)	0.67
	Basidiomycota	0.04 ± 0.01	0.46	4.04** (LDW, TRC, SERC, SCBI, WLF, HF)	1.01

*p<0.05
**p<0.01
***p<0.001

Figure S1: Genera that possessed a mean abundance over 0.5% of N-cycle community and change significantly in response to change in the abundance of ECM trees. Data is from the 54-plot gradient.

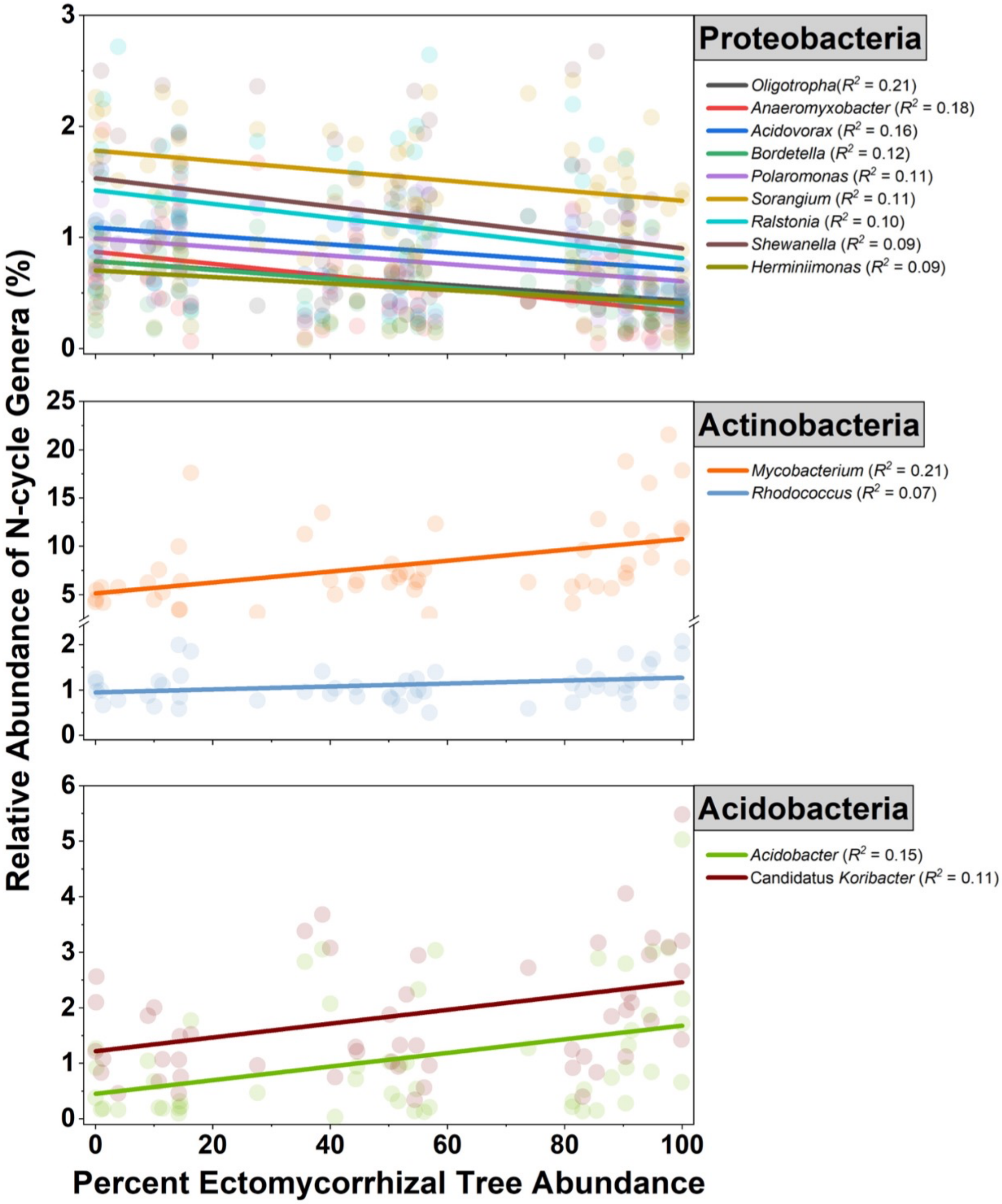

Figure S2: Linear regressions of N-cycle community metrics against the abundance of ectomycorrhizal trees.

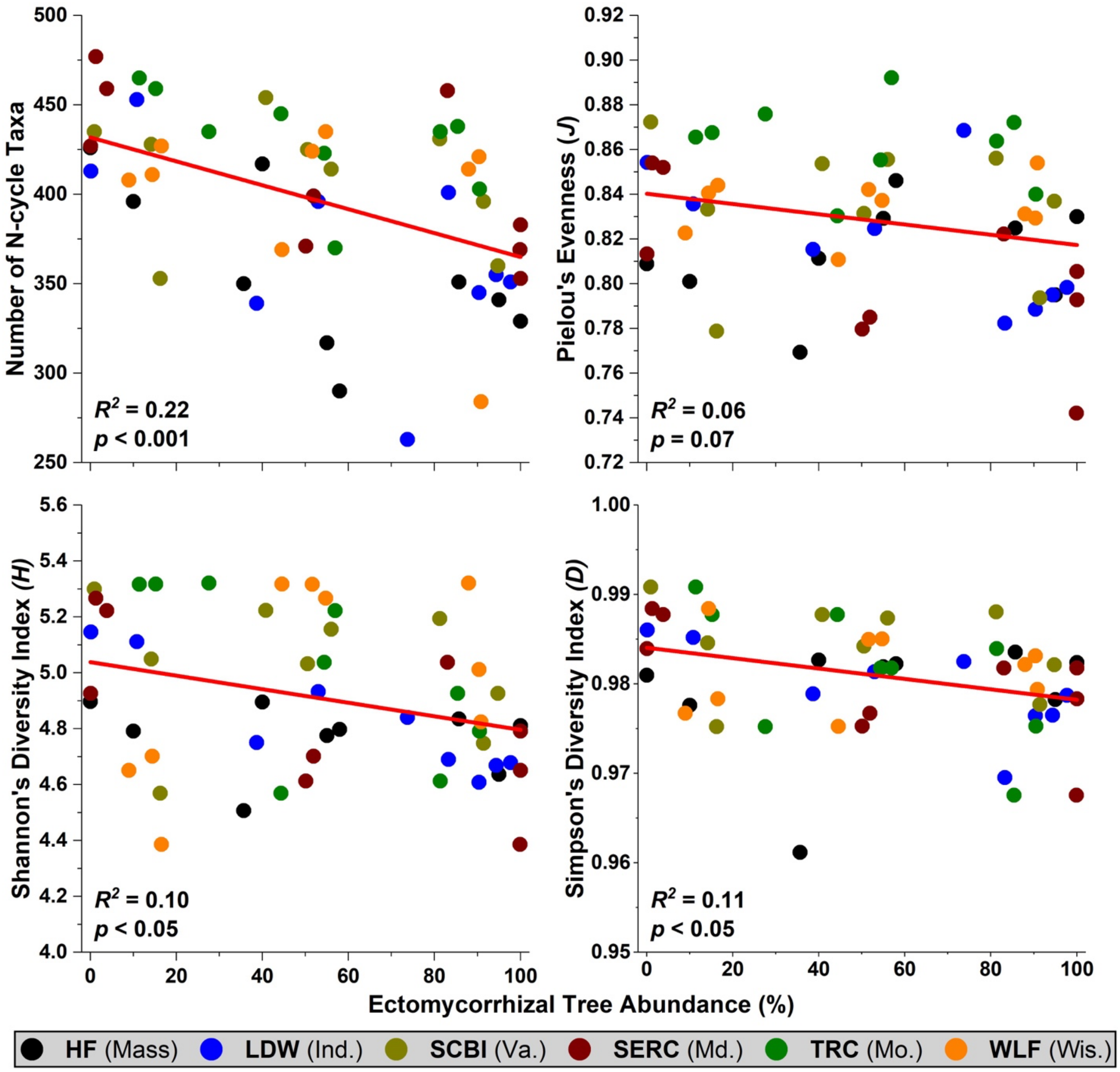

Figure S3: Proportion of N-cycle genes relative to all N-cycle genes in metagenomes from arbuscular (AM) and ectomycorrhizal (ECM) dominated plots at Moores Creek.

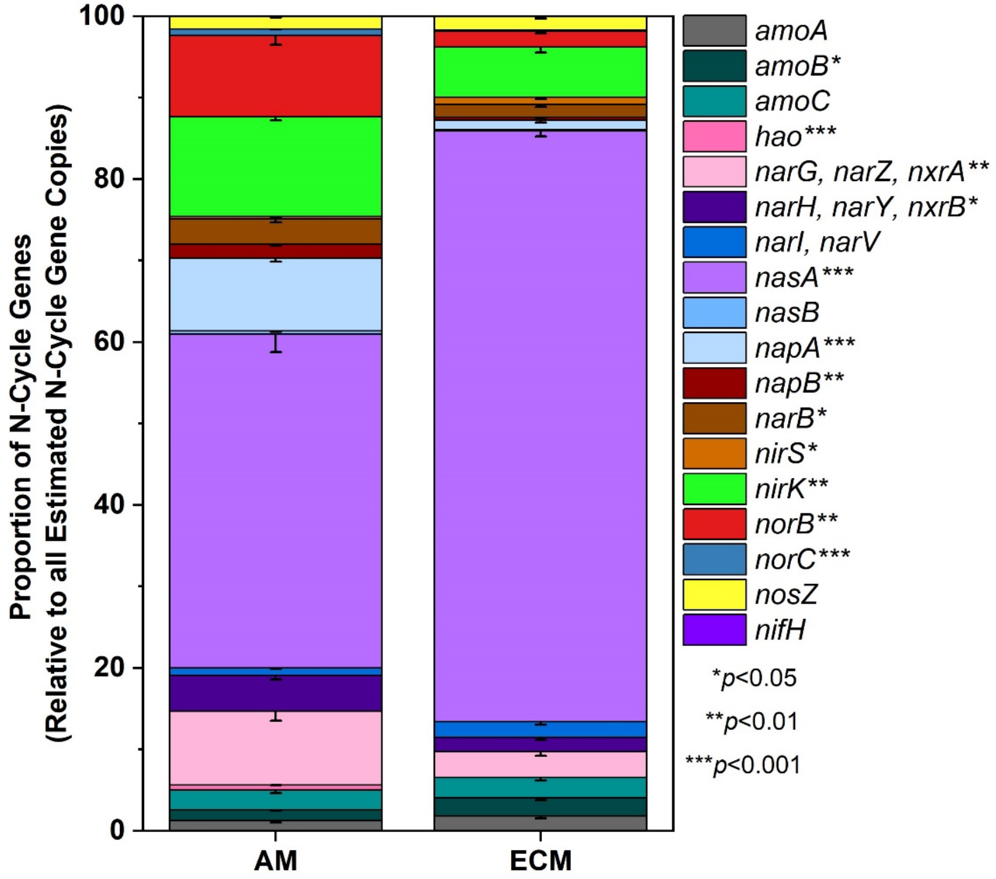

Figure S4: Soil nitrite + nitrate (NO_2^- + NO_3^-) concentrations under (1) environmental in situ conditions, (2) after 14-day aerobic (100% ultra-pure air) incubation, and (3) after a 14-day anaerobic (100% helium) incubation. Soil is from AM- and ECM dominated plots at Moores Creek. Each bar is the mean of N = 9.

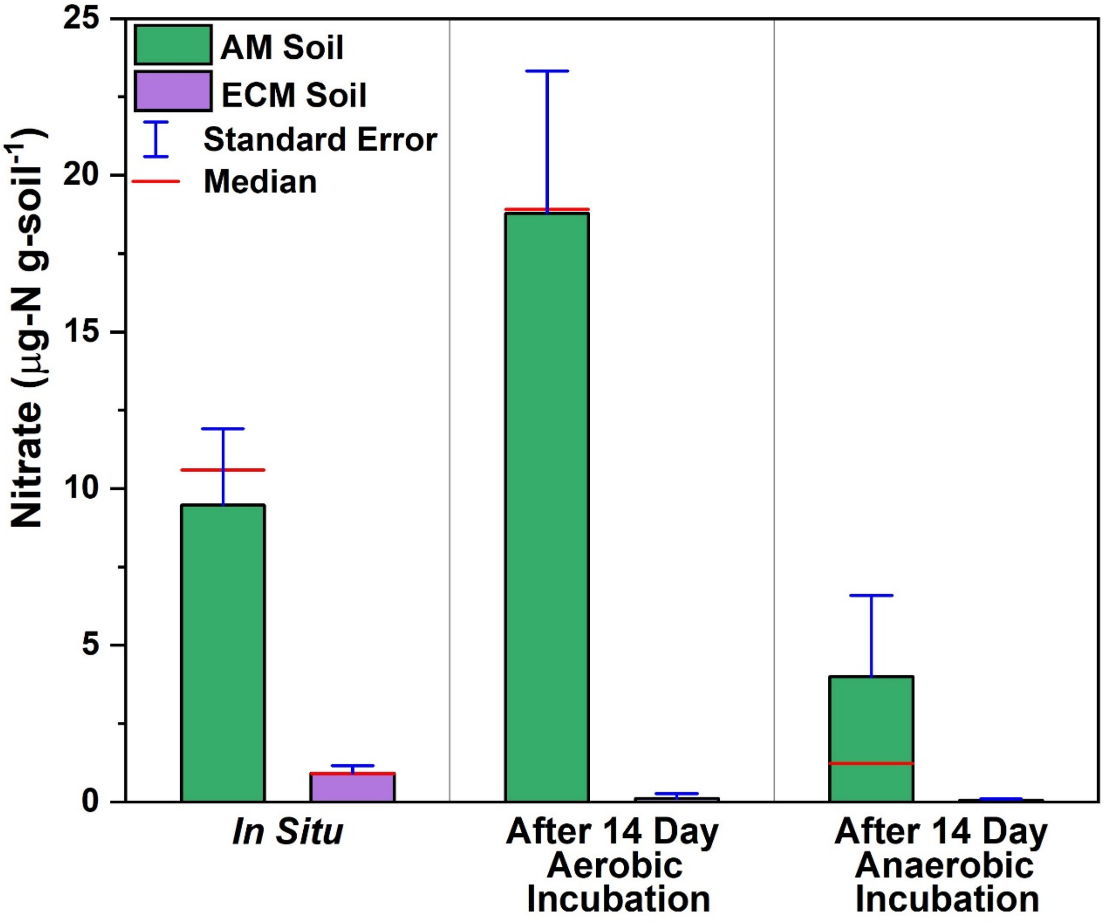

Figure S5: Results from mixed effects model where individual soil physicochemical properties were used as response variables and the percent of ectomycorrhizal trees was a fixed predictive variable. Geographic location (site) was included as a random variable. Model predictions shown adjacent to response graph. Soil used in this analysis are from the 54-plot gradient. Model fittings were only included for significant effects.

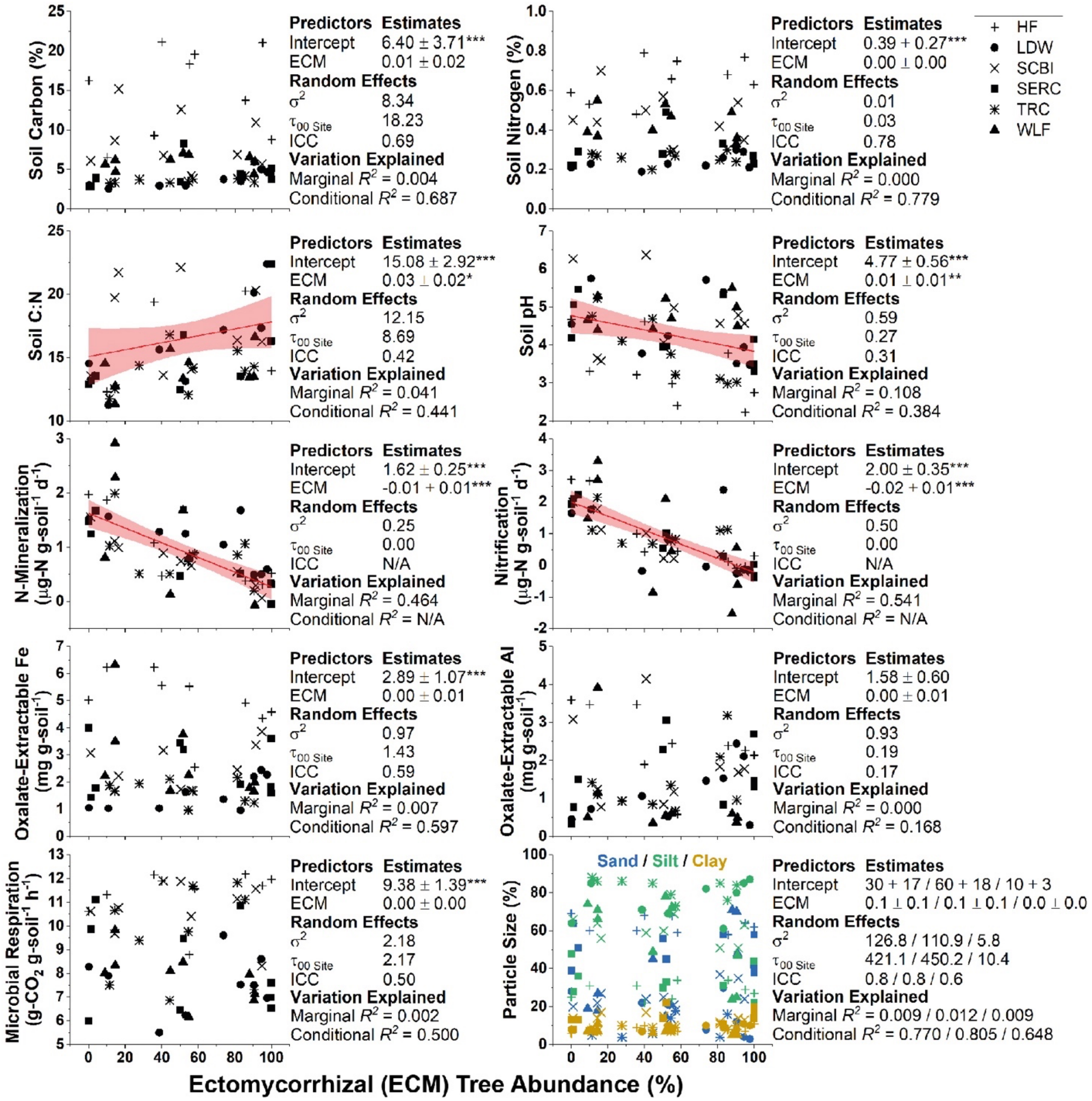

Figure S6: Transcript copies of key nitrogen cycle genes in AM- and ECM-dominated soil (15 g) following aerobic (100% ultra-pure air) and anaerobic (100% helium) incubations. AOA and AOB *amoA* = $NH_3 \rightarrow NH_2OH$; *nirS* + *nirK* = $NO_2^- \rightarrow NO$; *nosZ* = $N_2O \rightarrow N_2$. Limit of detection was 4×10^3 transcript copies per gram of soil. N = 9.

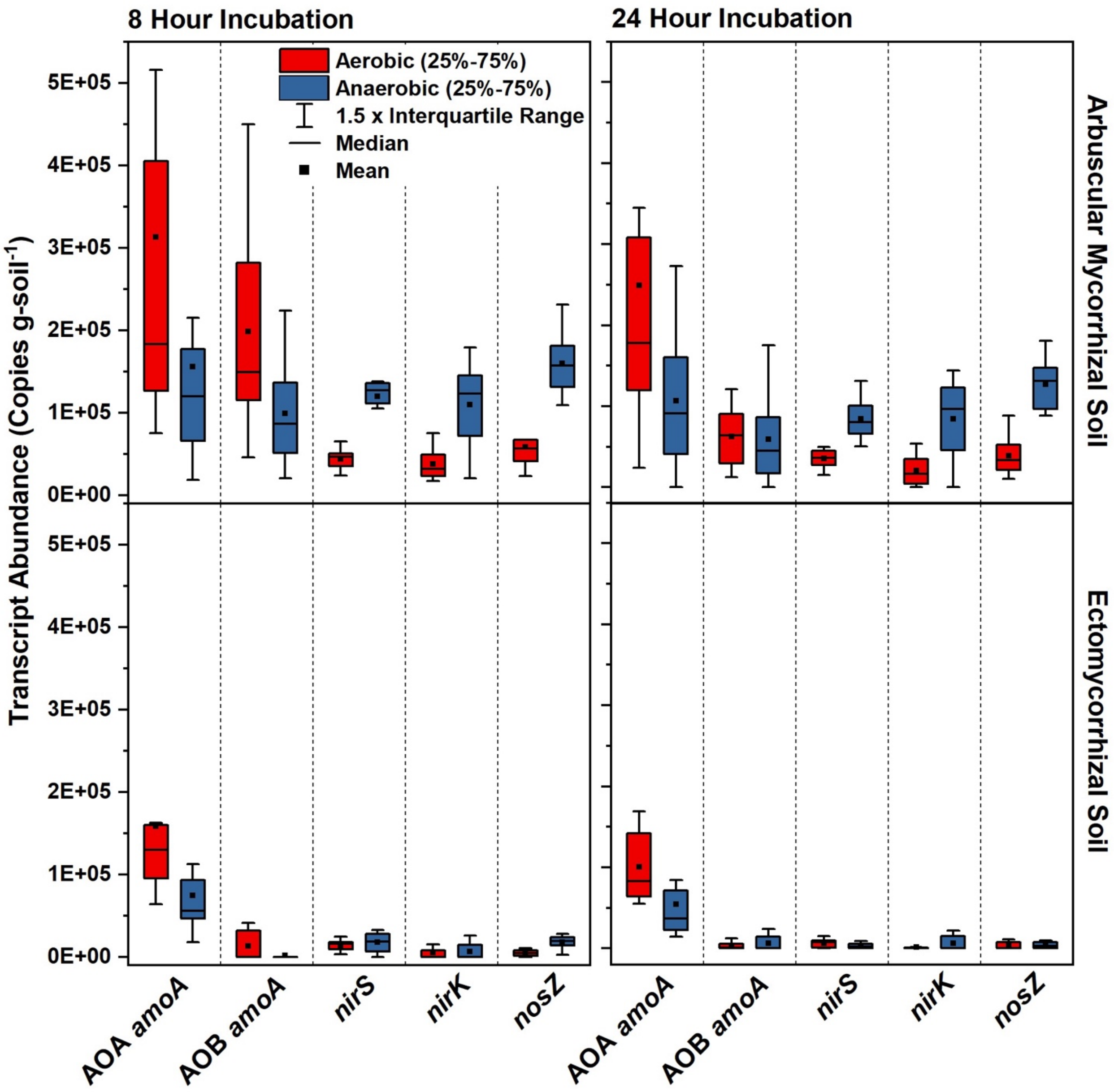

Figure S7: Soil ammonium concentration and rates of ammonification in arbuscular (AM) and ectomycorrhizal (ECM) dominated soil at Moores Creek.

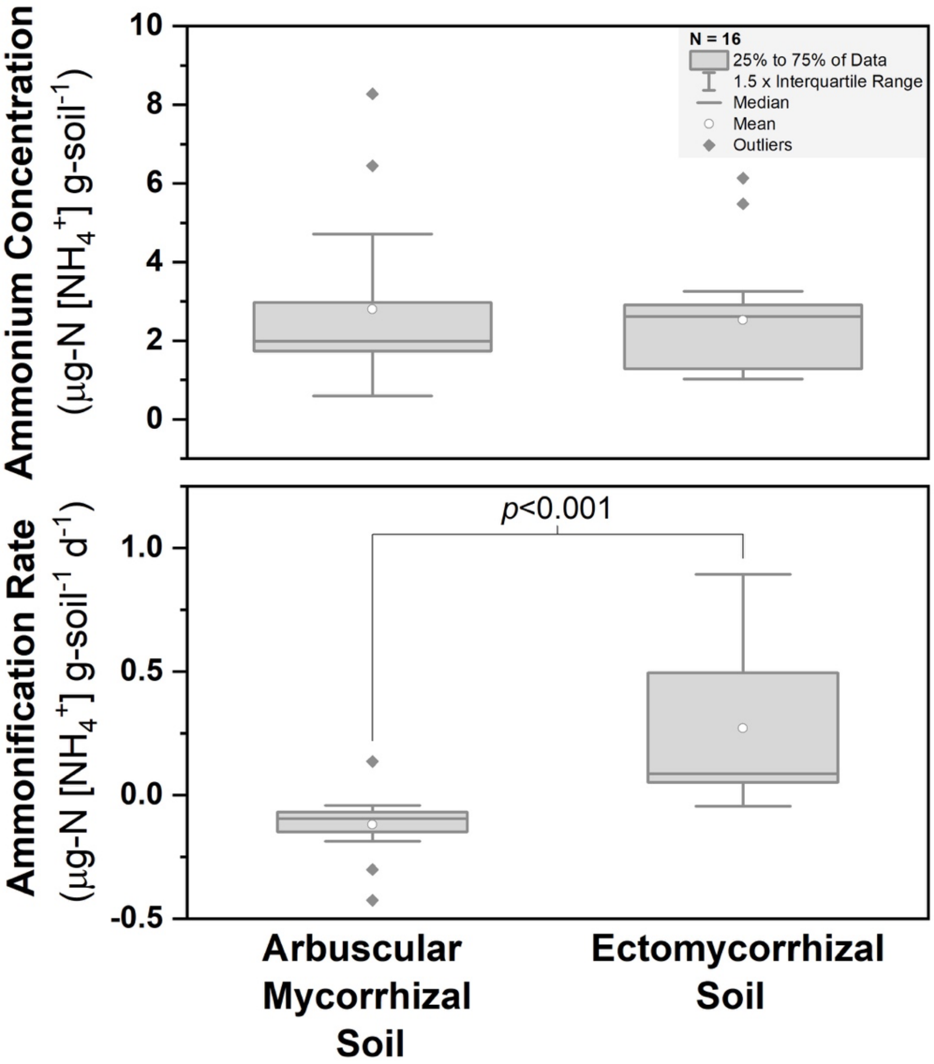

Figure S8: Time series showing denitrification activity (N_2O flux) and transcript copies of key denitrification genes following addition of nitrite to ECM soil. Aqueous nitrite (0.13 mg g-soil^{-1}) was added to ECM-dominated soil from Moores Creek, allowed to equilibrate for 15 minutes, and then analyzed for N_2O production under anaerobic conditions using the continuous flow system described above. For this experiment, roughly 10% of the gas flow onto the ECM-dominated soil contained acetylene (C_2H_2), resulting in a stable composition of 10% C_2H_2 in N_2 for the entirety of this analysis. Acetylene was added to inhibit the reduction of N_2O to N_2, thus allowing N_2O production to act as a proxy for denitrification activity. N_2O production in response to nitrite addition applied to steam sterilized ECM soil was also analyzed. To determine whether N_2O response was a function of microbial activity, we quantified the transcript activity of denitrifying genes (*nirS*, *nirK*, *norB*, and *nosZ*) in response to the addition of nitrite in ECM-dominated soil. In a series of anaerobic (100% helium atmosphere) destructive samples, RNA was extracted from ECM soil before nitrite addition and then every 15 min up to 2 h, and transcript abundance was quantified via RT-qPCR (details described in main text: 'RNA Extraction, cDNA Preparation, and qPCR'). Acetylene was not added during the measurement of transcripts. The solid blue line is the mean of N_2O flux and the highlighted region is the 95% confidence interval. The dashed brown line is the mean N_2O flux of steam-sterilized soil (N=3), which did not vary. Transcript points and bars are the mean ± standard deviation of three replicates.

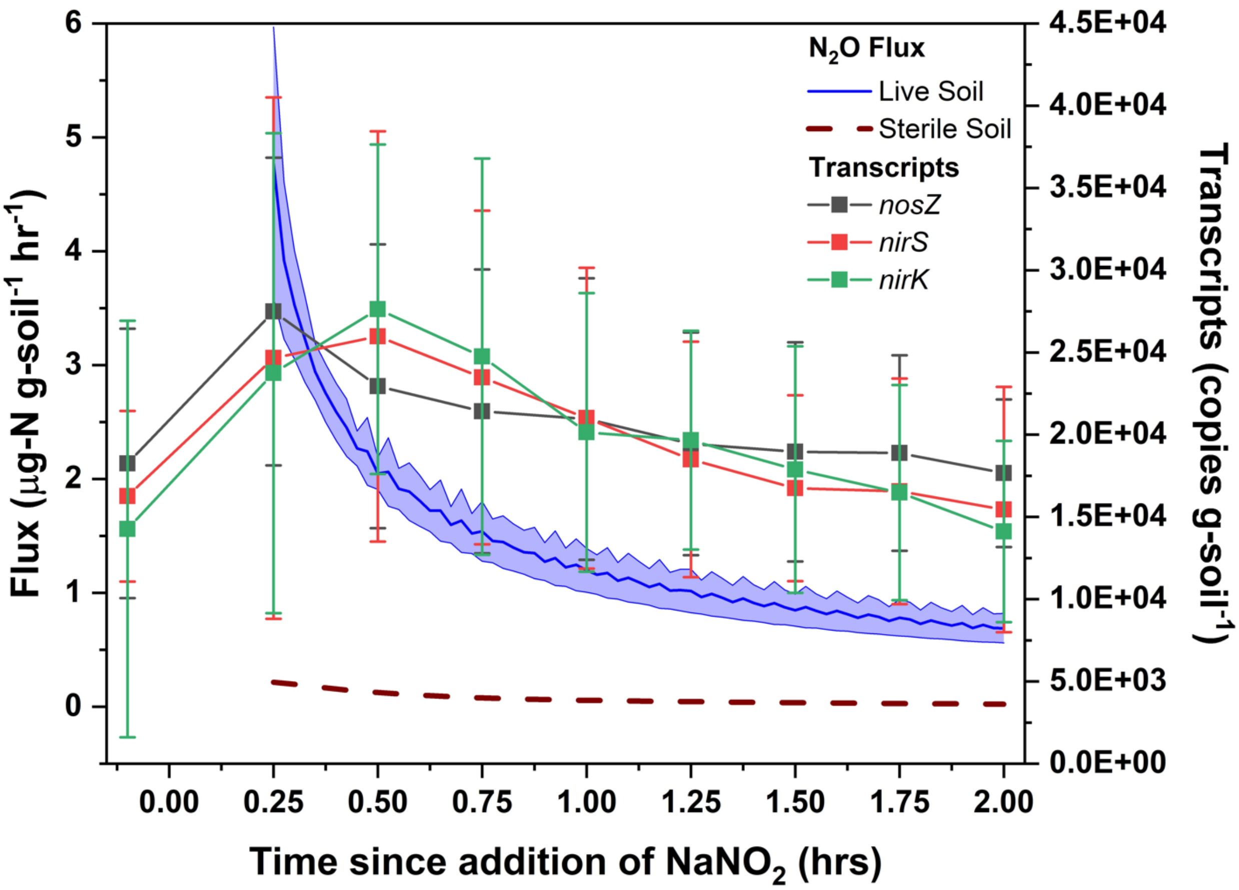

Supplement: Supplementary file 1 — Supplementary Material [file GCB-27-1068-s001.pdf]
